# Resting-State Electroencephalogram and Speech Perception in Young Children with Developmental Language Disorder

**DOI:** 10.3390/brainsci15030219

**Published:** 2025-02-20

**Authors:** Ana Campos, Rocio Loyola-Navarro, Claudia González, Paul Iverson

**Affiliations:** 1 UCL Ear Institute, University College London, London WC1X 8EE, UK; 2 Carrera de Fonoaudiología, Universidad San Sebastián, Santiago 7510157, Chile; 3 Departamento de Educación Diferencial, Universidad Metropolitana de Ciencias de la Educación, Santiago 8330014, Chile; rocio.loyola@ciae.uchile.cl; 4 Centro de Investigación Avanzada en Educación, Universidad de Chile, Santiago 7760197, Chile; 5 Departamento de Administración de Educación Municipal, Comuna de Independencia, Santiago 8380490, Chile; claudiag.flga@gmail.com; 6 Department of Speech, Hearing and Phonetic Sciences, University College London, London WC1N 1PF, UK; p.iverson@ucl.ac.uk

**Keywords:** DLD, resting-state EEG, oscillatory lateralisation, theta/alpha ratio

## Abstract

Background/Objectives: Endogenous oscillations reflect the spontaneous activity of brain networks involved in cognitive processes. In adults, endogenous activity across different bands correlates with, and can even predict, language and speech perception processing. However, it remains unclear how this activity develops in children with typical and atypical development. Methods: We investigated differences in resting-state EEG between preschoolers with developmental language disorder (DLD), their age-matched controls with typical language development (TLD), and a group of adults. Results: We observed significantly lower oscillatory power in adults than in children (*p* < 0.001 for all frequency bands), but no differences between the groups of children in power or hemispheric lateralisation, suggesting that oscillatory activity reflects differences in age, but not in language development. The only measure that differed between the children’s groups was theta/alpha band ratio (*p* = 0.004), which was significantly smaller in TLD than in DLD children, although this was an incidental finding. Behavioural results also did not fully align with previous research, as TLD children performed better in the filtered speech test (*p* = 0.01), but not in the speech-in-babble one, and behavioural test scores did not correlate with high-frequency oscillations, lateralisation indices, or band ratio measures. Conclusions: We discuss the suitability of these resting-state EEG measures to capture group-level differences between TLD/DLD preschoolers and the relevance of our findings for future studies investigating neural markers of typical and atypical language development.

## 1. Introduction

In the brain, synchronised neural activity underlies a wide range of cognitive skills, such as language and speech processing [1,2,3,4,5]. Neural oscillatory patterns vary depending on the task, but also occur as endogenous brain rhythms, which reflect spontaneous activation of cortical networks when the brain is awake but not actively engaged in a task. Distinct patterns of endogenous activity, detectable through a resting-state electroencephalogram (EEG), have been associated with different cognitive functions, including speech and language processing [6,7,8,9]. Even though the resting-state EEG does not reflect task-related activity, it captures endogenous rhythms that are thought to reflect the brain’s readiness and efficiency in language processing, even in the absence of explicit linguistic stimuli. Notably, research suggests that resting-state EEG patterns undergo developmental changes that may serve as predictors of language skills at later stages [10].

For these reasons, resting-state EEG measures have enormous potential as clinical indices of cognitive development [11,12]. Recording them requires no response or stimuli, making data collection easier than for experiments that involve a behavioural task, and also much shorter (usually requiring only 3–5 min of data). However, there is still little research on what changes in brain endogenous activity are relevant for language and cognitive development, and it is unclear if they play a role in atypical neurodevelopment (for example, in developmental language disorder).

The first aim of this study was to examine the potential relationship between endogenous oscillations and language typical/atypical development by comparing resting state EEG patterns between three groups of Spanish-speaking participants with different language skills: preschoolers with typical language development, preschoolers with developmental language disorder, and a group of neurotypical adults. A second aim was to determine if children’s resting-state EEG activity was associated with their performance in speech perception tests.

### 1.1. Resting State Spectral Power Measures and Language Processing

Changes in the brain’s endogenous activity can be quantified as variations in the spectral energy at different frequency bands. This measure is known as spectral power, and is calculated from the spectral decomposition of the raw EEG signal. Resting-state power is thought to reflect the baseline excitability of neural networks, encompassing those related to language and speech perception [13]. Thus, characterising resting-state EEG patterns and their changes along typical development is valuable for understanding how the brain’s intrinsic activity could be related to speech and language skills at different ages.

In adults, previous findings indicate that EEG resting-state power in different frequency bands is associated with performance in language or speech perception tasks. For example, Morillon et al. [14] observed that resting-state activity allowed spontaneous ‘tuning’ that predicted later performance in phoneme processing tasks, and was not observed for acoustically matched non-speech stimuli. A study by Breshears et al. [15] recording local field potentials in the superior temporal gyrus (STG) of patients undergoing brain surgery found that resting-state high gamma power (70–150 Hz) in the STG of both hemispheres correlated with performance in sentence perception tasks. Previously, a magnetoencephalography study by Houweling et al. [13] also found positive associations between resting-state power (mainly in the STG) and word-in-noise test performance, but with different hemispheric patterns; power was higher for beta (21–29 Hz) in the left hemisphere, and for gamma (30–40 Hz) in the right hemisphere. Oswald et al. [16] demonstrated that clusters of left-lateralised resting-state activity in the gamma band correlated with performance on verbal fluency tasks. All of these studies suggest that endogenous brain activity in different bands could provide an optimal neural context for speech perception, for example, by facilitating processing under adverse listening conditions. However, there is no compelling evidence of the relation between the developing brain resting-state activity and language skills.

Although it is known that resting-state patterns vary across the lifespan, there is no clarity on the functional implications of these variations for cognitive or language development. Changes in the patterns of resting-state power at different ages seem related to critical brain maturation processes and the development of global brain networks. For example, a decrease in broadband power has been associated with grey matter reduction due to synaptic pruning [17], cortico-cortical myelination, and changes in neurotransmission [18,19]. Others have proposed that increased theta power in infants and young children reflects a developmental state of enhanced synaptic plasticity that facilitates language acquisition [20].

Age-related changes in endogenous brain activity have been observed in rhythmic (periodic) neural activity affecting resting-state power measures [21]. Rhythmic neural activity becomes more consistent with age and more spatially focalized as a result of different neuromaturational processes and developmental cognitive changes [22]. In the EEG, this is reflected as a reduction in broadband (or ‘absolute’) power, an increase in oscillatory coherence, a power redistribution, and changes in the topography, peaks, and boundaries of different frequency bands [18,19,23,24]. Thus, canonical frequency bands in adults are not necessarily equivalent to those observed in children [21], which children bands tending to be lower in their ranges and peaks [25], although they still show the characteristic 1/f EEG structure (power reduction as a function of frequency). Similarly, neural background noise decreases from childhood to adolescence [26,27], and from adolescence to adulthood [28], also reflecting neuromaturation and facilitating the resting-state EEG analysis.

Maturational changes in the resting-state EEG power involve an age-related decrease in spectral energy for low frequencies and an increase for high frequencies [25,29,30,31]. For example, studies by Yordanova and Kolev [31] and by Perone et al. [32] described a decrease in theta (4–7 Hz) and an increase in alpha (~7–13 Hz) resting-state power from the age of 6 to 11 years, with a continuous power reduction during adolescence for frequencies below 8 Hz. According to Uhlhaas et al. [19], gamma-band oscillations emerge during early childhood and show developmental changes until adulthood, but the direction of these changes seems unclear. A large-scale study by Takano and Ogawa [33] reported a steep increase in frontocentral resting-state gamma power (35–45 Hz) in young children between the ages of 3 and 4 years, but with later stabilisation from the ages of 4 to 12 years. On the contrary, another large study by Tierney et al. [17] demonstrated less frontal gamma power (31 to 50 Hz) in older participants than in young children (3–5 years), with strong negative correlations between resting gamma power and age from early childhood to adulthood.

Developmental changes in spectral power also seem to underpin cognitive and language development. For example, Guo et al. [34] proposed that higher resting-state frontal gamma power supports attentional and working memory processing, whereas Kwok et al. [35] suggested that reduced alpha power reflected greater attentional control, positively influencing performance in language tasks. Nevertheless, very few studies have investigated the relationship between endogenous neural activity, language skills, and speech perception development in young children. Although behavioural evidence consistently shows that children’s speech perception improves with age [36], as confirmed by increasingly better performance in speech-in-noise discrimination tasks as children grow up [37,38], there is less evidence about how these skills are related to changes in resting-state EEG patterns.

In infants and young children with typical language development, a few studies indicate that resting-state power significantly correlates with and even predicts language skills. Gou et al. [34] found that frontal resting gamma power (31–50 Hz) at 16, 24, and 36 months old positively correlated with performance in several language tests (non-word repetition, PLS-3 and CELF-P sentence structure subtests) at the later ages of 4 and 5 years. Likewise, a longitudinal study by Cantiani et al. [39] reported that increased left-lateralised resting-state frontal gamma power measured at the age of 6 months correlated with better language outcomes at the age of 24 months. For the alpha band (7–10 Hz), Kwok et al. [35] found that resting-state power in children (4–6 years) inversely correlated with performance in language tests (CELF-5), suggesting that alpha activity could reflect increased neural inhibition and less excitability related to selective attentional control.

At later ages, resting-state theta power seems inversely correlated with language skills, whereas beta power seems directly correlated. A study by Lum et al. [40] in children around ten years old reported that resting-state theta power negatively correlated with sentence repetition performance. Similarly, a longitudinal study by Meng et al. [29] reported that reduced theta power predicted better expressive vocabulary outcomes at the ages of 9 and 11 years. In contrast, increases in beta power from the age of 7 to 9 years predicted better receptive vocabulary skills at age 11. These findings suggest that resting-state oscillatory power changes occur throughout typical brain maturation, and may serve as clinical indexes of language and cognitive development. For example, Cantiani et al. [39] proposed that resting gamma oscillation patterns could be an early screening tool for infants at risk of language deficits. However, these studies have not established which brain networks generate each neural activity pattern or what their specific functional role is (i.e., attentional or language).

Although the evidence indicates that spontaneous brain activity plays an essential role in speech perception and language development, there is strikingly little evidence about resting-state power in children with developmental language disorder, despite this condition affecting as many as 7.5% of school-age children with a high impact on their ability to produce and comprehend their native language [41]. In other neurodevelopmental disorders, such as dyslexia and autism spectrum disorder, research suggests that atypical resting-state patterns underlie cognitive and language deficits (e.g., see [42,43] for studies in autism and dyslexia, respectively), for example, because of atypical cortical maturation or imbalances between neural excitatory and inhibitory activity [44]. Thus, it is plausible that atypical resting-state patterns could also occur in developmental language disorder, but there is little evidence of oscillatory differences in this disorder.

To our knowledge, the only study examining the relationship between the maturation of resting-state oscillations and language abilities in infants at risk of developmental language disorder is by Benasich et al. [45] They compared resting-state patterns in infants with a family history of developmental language disorder in a first-degree relative and their age-matched controls, testing them longitudinally at the ages of 16, 24, and 36 months. The authors found that the group with family history of developmental language disorder showed consistently lower gamma power over frontal brain regions than the controls. Moreover, frontal gamma power strongly correlated with language and cognitive skills at all ages, and children with higher gamma power showed more mature inhibitory control and attention-shifting skills. The authors concluded that the emergence of high-frequency neural synchrony might be critical for cognitive and linguistic development. However, these findings in infants have not been replicated in children or in groups with a clinical diagnosis of developmental language disorder, and not only at family risk of presenting it.

### 1.2. Hemispheric Asymmetries in Endogenous Oscillations

The asymmetry (or lateralisation) of oscillations refers to the differences in the activity patterns at different frequencies between the cerebral hemispheres. Hemispheric asymmetries are a crucial feature of the brain’s functional organisation, and they are especially relevant for speech perception. In adults, a large body of evidence indicates that both speech-evoked and endogenous oscillations are asymmetrical, and that this asymmetry is relevant for language processing [4,5].

A predominant explanation of the role of oscillatory lateralisation in speech processing is the Asymmetric Sampling in Time Hypothesis [46,47]. This theory states that non-primary auditory areas show differences (or biases) in the patterns of resting-state oscillations between hemispheres. These biases are related to differences in the distribution of the centre frequency at which neuronal ensembles synchronise spontaneously. In the right hemisphere, neural ensembles are skewed towards synchronising at a theta rate (3–7 Hz), and in the left hemisphere, towards low-gamma (20–50 Hz) frequencies [48]. During speech perception, this resting-state asymmetry would “prime” the brain for sampling different features on each hemisphere. The left hemisphere would be primed for extracting information over shorter intervals (20–50 ms) and processing fast acoustic changes, such as the transitions between consonant and vowel sounds, whereas the right hemisphere would be primed for sampling speech over longer time windows (~150–300 ms), required for prosodic processing [48,49]. However, the Asymmetric Sampling in Time Hypothesis is not specific about the developmental trajectories of these resting-state asymmetries.

Although there is agreement that the establishment of oscillatory hemispheric asymmetries represents a neuromaturational milestone for language acquisition, there is no consensus about its timing. Some studies have shown that infants as young as newborns exhibit left-lateralised neural responses to speech sounds [50], suggesting that the basic neural mechanisms for speech processing are present early in life, but it is unclear at what age resting-state oscillations become asymmetric. For example, Thompson et al. [51] conducted one of the few studies in typically developing children between 3 and 4.9 years old examining gamma resting-state oscillations and speech perception. In 3 year-old children, they observed a consistent leftward asymmetry in the low gamma range (20–50 Hz), but no rightward asymmetry in the theta band (3–7 Hz), suggesting that the hemispheric oscillatory specialisation develops later in the right than in the left hemisphere, and for low than high frequencies. This study also found that children with more pronounced resting gamma left-lateralisation showed better performance on speech-in-noise tests. Thompson et al.’s [51] findings support the Asymmetric Sampling in Time Hypothesis by linking greater resting-state asymmetry in high frequencies with better speech processing under challenging conditions, although without information about the specific developmental aspects.

Regarding developmental language disorder, a popular hypothesis is that atypical oscillatory lateralisation and lack of leftward asymmetry may play a crucial role in this condition (see [52] for a review). However, most of the findings about language lateralisation in developmental language disorder come from haemodynamic studies (e.g., fMRI), as in [53,54], and have not been linked to resting-state oscillations. For example, it has not been determined whether there is a lack of oscillatory priming on each hemisphere in developmental language disorder, as could be expected under the Asymmetric Sampling in Time Hypothesis. Moreover, many studies have used handedness as a behavioural proxy of language brain lateralisation, which may lack reliability [55] and provide inconsistent evidence about the role of atypical hemispheric lateralisation in developmental language disorder. This was pointed out by a large-scale fMRI study in twins by Wilson and Bishop [56] that did not find any evidence of greater atypical lateralisation in children with developmental language than in controls, concluding that the lack of a functional asymmetry in the brain may not necessarily involve poor language development.

### 1.3. The Current Study

To date, there is no clarity regarding the developmental patterns of endogenous oscillations or the association between resting-state measures and specific language or cognitive skills. Even though the Asymmetric Sampling in Time Hypothesis provides a framework for understanding the role of oscillatory lateralisation in speech processing, most of the evidence supporting it comes from studies of adults, and not from children, and no study has explored the role of resting-state patterns in preschoolers with developmental language disorder. So far, we do not know whether these children exhibit delayed, altered, or typical resting-state patterns, and what the significance of these patterns is for this condition’s behavioural symptoms. Understanding these aspects is extremely important, given the current need for objective clinical markers that could help to improve the identification of children at risk of developmental language disorder at early ages. Considering this gap in knowledge, we conducted a resting-state EEG study in children with developmental language disorder to determine if previous findings in typically developing children could be confirmed in this clinical group. Namely, we looked at an association between resting frontal gamma power and language skills (as in [34]) and between high-frequency leftward asymmetry and speech-in-babble performance (as in [51]) or language status (as in [45]). However, as the amplitude of gamma oscillations is small and hard to measure because of the 1/f spectral power distribution and the low signal-to-noise ratio in young children’s EEG, we also examined activity for the theta, alpha, and beta bands.

Our first goal was to characterise resting-state band power at lateralisation of oscillations at different frequencies in young children with typical language development and developmental language disorder, and to compare their responses to those observed in adults. A second goal was to compare the performance of both groups of children in speech perception tests and determine whether there was a relationship between behavioural and resting-state EEG measures. Thus, we addressed the following research questions: What are the patterns of resting-state power and lateralisation of oscillations at different bands for each group of participants? Are there any differences between the typical language development, developmental language disorder, and adult groups in resting-state power and lateralisation at different frequency bands? In children with typical language development and developmental language disorder, are any of the EEG variables associated with speech perception tests scores?

Considering that the literature on resting-state oscillations in typically developing preschoolers is scarce and almost inexistent for children with developmental language disorder, we aimed to examine whether previous findings could be extended to these populations. Due to the limited existing evidence about resting-state EEG patterns in preschoolers, our approach was exploratory, and focused on highlighting potential areas for further investigation that could help in the generation of future hypothesis. The primary hypothesis was that resting-state measures and language status at the group level would be positively associated. Thus, we predicted that typically developing children would exhibit (i) greater resting frontal gamma power, and (ii) stronger high-frequency asymmetry than children with developmental language disorder. In adults, we expected (i) reduced gamma power because of brain maturational changes (e.g., [18,19]), and (ii) no differences with children in the strength of the leftward asymmetry, as this should be already established by the age of our sample [51,54]. The secondary hypothesis was that speech perception skills would show differences based on children’s language status (typical language development/developmental language disorder), but also an association with EEG lateralisation measures, as reported by Thompson et al. [51]. Thus, we expected typical language development children to perform significantly better than the developmental language disorder group in speech perception tests. Test scores would be associated with resting-state gamma band power and high-frequency lateralisation indices. Finally, although we had no a priori hypothesis for the theta, alpha, and beta bands, we expected the typical 1/f structure in all groups, but with a smaller amplitude in adults because of the age-related power decrease [25].

## 2. Materials and Methods

### 2.1. Participants

Twenty-seven monolingual Spanish-speaking children and eighteen adults participated in this study, all of whom were screened for normal hearing. Children were recruited from the same public preschool located in Santiago, Chile, and divided into two groups according to their language status: one group of sixteen children with a previous clinical diagnosis of expressive–receptive developmental language disorder (6 female, mean age = 5.2 years, SD = 0.33, age range 4.9–5.7 years), and a group of eleven age-matched controls with typical language development (7 female, mean age = 5.2 years, SD = 0.23, age range 4.10–5.6 years). Mann–Whitney U tests indicate that the children’s groups did not differ in age (U = 86.5, *p* = 0.960, with a small effect size indicated by rank biserial correlation coefficient *r* = 0.02) or hearing level (PTA left ear: U = 86, *p* = 0.927, with a small effect size *r* = 0.02; PTA right ear: U = 66, *p* = 0.230, with a small effect size *r* = 0.25), determined via play audiometry at 500, 1000, 2000, and 4000 Hz (see Supplementary Materials Table S1 for age and hearing level descriptives and normality/variance homogeneity tests, and Figure S1 for screening variable distributions). During the screening, children were excluded from the study if they were not able to complete the audiometry and a non-verbal task, or if their hearing thresholds (PTA) were above 25 dB on either ear. Only children without a significant medical history (any serious illness or neurodevelopmental condition other than DLD, hearing problems, prematurity, cognitive or motor delays, or mental health issues) were invited to participate in this study. This was determined through a parent survey that also collected information about the children’s developmental history, language background, and parent’s education level.

Recruiting the children from the same preschool was essential to control for socioeconomic (SES) and educational factors that introduce variability, and are known to affect cognitive and language development [57], thus potentially affecting resting-state measures. Sampling from the same school ensured that all children lived within the geographical school catchment area and received similar education, which only differed in the specialised speech and language therapy support provided by the school to the group with developmental language disorder. Children’s SES was also controlled through parental education, ensuring all parents/carers had completed their mandatory education at least until the age of eighteen years, according to Chilean legislation. For more details about demographics, screening data, recruitment, and other EEG studies in this sample, see Campos et al. [58].

To allow for age-related comparisons between children and adults, we included data from eighteen adults (mean age = 33.7 years, SD = 4.9, age range = 24.8–44.9, 11 female) from a previous validation study conducted six months earlier (Campos et al., in prep). All adults were native Chilean Spanish-speaking adults who lived in London (UK) and were recruited via social media groups. They were all born in Chile, with a high proportion of Spanish use in their daily life (e.g., they currently use Spanish as their first language at home). All of them had English as a second language with different levels of proficiency, but none of them were early bilinguals (e.g., they did not speak or were spoken to regularly in any language other than Spanish before the age of five years). Only three adults reported living in an English-speaking country for more than five years, and six of them did not consider themselves fluent enough in English to be bilingual. None of the adult participants reported a history of hearing loss, neurological or psychiatric conditions, or learning or language difficulties, as determined by an online screening survey, and all of them were able to complete a non-verbal task [58]. The adult’s hearing levels were assessed via air-conduction audiometry to confirm that they presented pure tone average (PTA) thresholds ≤ 20 dB for both ears at octave frequencies from 500 to 4000 Hz and a threshold of ≤25 dB at any given frequency from 250 to 8000 Hz.

### 2.2. Speech Perception Measures

Speech perception tests inform about the ability to extract meaningful speech cues from complex acoustic environments. For this study, we considered two low-redundancy monoaural tests [59], a speech-in-babble and a filtered speech test, justifying their selection on two criteria: (i) the tests must be supported by robust evidence demonstrating their reliability for assessing speech perception skills, and (ii) the tests must include normative data relevant to the population being evaluated [59].

The two tests used in this study have been widely studied in the literature [59], and were obtained from the “Santiago Auditory Processing Battery” [60], which has normative data for Chilean children. In this battery, each subtest consists of fifty Spanish monosyllabic words divided into two lists of 20 stimuli (one list for each ear, plus two practice items) delivered via headphones. All stimuli in this battery are balanced in their linguistic frequency and age of acquisition for Chilean Spanish (Appendix B).

The speech-in-babble subtest requires participants to recognise words embedded in multi-talker babble, both presented in the same ear at 40 dB SL with a non-adaptive, fixed signal-to-noise-ratio equal to 0. This test simulates real-world listening scenarios, and assesses an individual’s ability to segregate target speech in the presence of competing talk. We decided to use a speech-in-babble test, as we wanted to corroborate previous findings by Thompson et al. [51] about the association between resting-state EEG measures in preschoolers and their speech perception skills in background noise.

The filtered speech subtest consists of recognising low-pass-filtered monosyllables, in this case at 1500 Hz presented at 50 dB SL. These tests manipulate the acoustic characteristics of speech to assess the ability to process spoken stimuli with degraded spectral or temporal cues. We chose this test because it attenuates frequencies above 1500 Hz, which are important cues for speech discrimination increasing the demand on the listener’s phonological processing skills due to insufficient acoustic information. We expected that typically developing children would be more resilient to these manipulations than children with developmental language disorder, thus performing better in this test, which may be reflected in the EEG measures.

For each speech perception subtest, we asked children to repeat a list of target words, presented one after another, with each correct answer scoring 5%. According to Chilean normative studies, age-expected values for these tests in typically language development children are over 60% (e.g., [61]). The order of the subtests and starting ear were randomly determined to avoid the potential effects of the presentation order. Before the tests, children received a Chilean articulatory screening ([62] see Appendix C) to check their phonemic repertoire and avoid confounds when scoring the speech perception tests.

### 2.3. Procedures

Data collection involved three sessions for children and one session for adults. After a careful pre-selection by the school Speech and Language Therapist, children who meet the inclusion criteria and did not meet the exclusion criteria were invited to participate in the study (25 children per group). After their parents completed a family history and developmental questionnaire, children who did not present any other comorbid condition attended a first screening session at their school. This consisted of some hearing tests (otoscopy and play audiometry), and completion of a manual task to check the children’s ability to follow instructions. Three months later, children who passed these screenings (*n* = 17 for the typically developing and *n* = 20 for the developmental language disorder group) were invited to our lab for the second and third testing sessions to collect EEG and behavioural data (speech perception tests), respectively. The final number of children who completed the EEG session was *n* = 16 for the developmental language disorder and *n* = 11 for the control group, whereas for the behavioural session, the final sample was *n* = 11 and *n* = 8, respectively.

Adult EEG testing was conducted at University College London, Infant and Child Language Lab, whereas child EEG testing was conducted at Universidad de Chile, Neurosistemas Lab. In both laboratories, the EEG testing room was electrically shielded and acoustically isolated from the rest of the facilities, reducing external electrical interference and distracting stimuli. During the EEG session, children sat still and quietly next to their parents or carers. According to previous studies [51], eyes open resting-state EEG was recorded continuously for three minutes, as this would provide enough data for group-level comparisons while having a short recording time. To limit eye movements, children had to fix their gaze in an 8.7-inch tablet that presented a black screen, placed 100 cm in front of them at their eye’s level. Keeping the fatigue levels to a minimum was the main reason to limit the EEG recording time to no more than three minutes, as having the children staring at a black screen for longer than this would increase the possibility of boredom, tiredness, or sleepiness. The EEG was recorded with a 32-channel Biosemi Active Two system at a 2048 Hz sampling rate in 10–20 electrode montage, with DC offsets levels kept under 30 µV. Vertical and horizontal electro-oculogram were recorded in the right supraorbital area and right eye canthus, respectively. All of the other procedures were the same as those in Campos et al. [58].

To control the children’s arousal levels during the EEG recordings, we adopted different procedures. Firstly, all testing was conducted during the daytime, in hours during children are active and awake and all parents confirmed that their child had a proper sleep the previous night. Children in both groups were randomly assigned to six different two-hour EEG testing slots covering different hours in the morning and afternoon, with no significant association between the groups and their allocated testing slots, as indicated by Fisher’s exact test (*p* = 0.827, Cramer’s V = 0.374; see Supplementary Materials, Tables S4 and S5). Secondly, to avoid somnolence during the EEG recording, the testing room temperature was set at 18 degrees Celsius, and the lights were not fully dimmed. Thirdly, after setting up the EEG cap and electrodes, the examiners (all experienced paediatric SLTs) verbally assessed that the children were alert and able to interact with them, by asking questions or chatting about the child’s interests. The presence of fatigue was verbally checked by asking the children if they were tired or happy to continue, as per ethical requirements. Finally, arousal was also monitored online through the EEG recording software, Actiview (©). Arousal decrease is easily identifiable in the EEG raw traces by the presence of longer, more frequent blinks and larger occipital alpha waves; none of this was observed in the participants during the EEG recording.

### 2.4. EEG Preprocessing

EEG analysis was performed with Matlab 2016–2022a, EEGLab [63], and Fieldtrip [64]. During data collection, the EEG was referenced online to the CMS active electrode, as children did not tolerate the mastoid electrodes well. For each participant, the EEG was downsampled to 500 Hz and referenced offline to electrode Cz. Electrode Cz was chosen to improve the data quality, allowing us to retain more epochs than the average reference, as data are usually noisier in children [65]. We applied a high-pass Butterworth IIR filter (non-causal, zero-phase shift, second order) with a cut-off of 0.1 Hz to reduce slow drifts and improve the ICA decomposition. After applying an initial automatic detection threshold of 600 µV peak-to-peak to remove excessively large artefacts, the continuous EEG was visually inspected by experienced researchers to detect and remove bad channels and noise-contaminated data portions. Visual inspection aimed to identify and manually remove sections of the data contaminated with artifacts such as large eye-blinks, ocular movements, muscle activity, or electrode noise, before semi-automated cleaning via Independent Component Analysis (ICA). The mean percentage of channel rejection for adults was 13.79% (SD = 5.5, min-max = 3.1–21.9%), with 10.53% (SD = 3.2, min-max = 6.3–15.6%) for the typically developing group and 10.76% (SD = 5.08, min-max = 6.3–25%) for the group with developmental language disorder. Out of the 45 participants, 33 (73.33%) of them had ≤15% of rejected channels; 10 participants (22.22%) had between 15 and 20%, whereas only 2 participants (4.44%, one adult and one child in the language-impaired group) had between 21 and 25% of their channels rejected. ICA was performed in EEGLab [63] to eliminate remaining eye blinks, eye movements, muscular artefacts, and activity from other non-cortical sources. After cleaning, the removed channels were spherically interpolated, and data were re-referenced to the average.

The preprocessed EEG was segmented into fifty-seven 2 s epochs (1000 samples) with 50% overlap, windowed with a Hanning taper to attenuate the edges and avoid ridge artefacts (see Figure S2 for example waveforms). This epoch length was chosen to ensure that low-frequency activity would not be affected, as discussed by Thompson et al. [51]. For each epoch, we computed the frequency spectrum at each channel between 2 and 60 Hz in steps of 0.5 Hz using a Fast Fourier Transform, resulting in 116 linearly spaced frequencies with a 0.5 Hz frequency resolution. To avoid distortions resulting from the filter cut-off and line noise artefacts, band power analysis was restricted to the 2–45 Hz range (92 frequencies) and calculated across the following frontal and central channels: Fp1-2, AF3-4, F7-8, F3-4, FC1-2, FC5-6, and Fz. Then, power was binned into the theta (3–7 Hz), alpha (8–12 Hz), beta (13–25 Hz), and gamma (25–45 Hz) bands and averaged within each band (e.g., as in [49]) and group.

The lateralisation of oscillations was calculated as in previous studies (e.g., Thompson et al., [51]), dividing the EEG channels into two sets: left (FP1, AF3, F3, F7, FC1, FC5, T7, C3, CP1, CP5, P3, P7, PO3, O1), and right (FP2, AF4, F4, F8, FC2, FC6, C4, T8, CP2, CP6, P4, P8, PO4, O2), excluding the midline electrodes. Spectral power was averaged within each electrode set between 2 and 45 Hz, and a “laterality index” (LI) was calculated at each frequency with the following formula: LI = Absolute Power (Right − Left)/Absolute Power (Right + Left). A number less than zero indicated a bias of oscillations towards the left hemisphere, and higher than zero towards the right hemisphere. For each participant, the laterality indices were averaged into a low-frequency bin (3–7 Hz) for the theta range and a high-frequency bin (20–45 Hz).

### 2.5. Design

This study was observational, and involved within- and between-group analysis. The independent variable was language status, operationalized as the “Group” category, with three levels: typical language development, developmental language disorder, and adults. The dependent variables were all continuous and included EEG measures of: (i) average band power (in µV^2^) at the theta, alpha, beta and gamma bands; (ii) oscillatory lateralisation (positive, negative, or neutral indices); and (iii) the percentage of correct responses for the speech in noise and filtered speech tests.

### 2.6. Statistical Analysis

Statistical analyses were performed with Matlab 2016–2022a and SPSS 22–29. When the assumptions for linear methods were unmet, non-parametric tests were preferred over bootstrapping or permutation methods, as the former performed better with small sample sizes.

Within groups, we expected inherent differences between frequencies for the average band power because of the 1/f spectral structure. However, our focus of interest was determining between-group differences at each frequency range. Thus, to test our primary hypothesis, we conducted planned comparisons between the groups but separately for each band or laterality measure using one-way ANOVA or Kruskal–Wallis tests, when parametric assumptions were not met (see Appendix A and Appendix B for data distributions and assumptions checks, respectively). To avoid inflating the family-wise error because of multiple testing, we used Bonferroni-corrected alpha levels for all the planned comparisons on band-power and lateralisation measures.

To test the secondary hypothesis, we compared the performance for each speech perception test only between the groups of children, as these tests were not conducted in the adult group due to practical reasons (permission to use the adult auditory processing test battery was not granted at the time of testing) and also because normal hearing adults usually perform at ceiling level in these tests. Firstly, we examined the between-group differences in the test scores using independent samples *t*-tests or Mann–Whitney’s U tests, when parametric assumptions were not met. Secondly, we explored the association between speech perception and EEG measures (gamma power and high frequency asymmetry) using Pearson’s correlation or Spearman’s rank analysis when parametric assumptions were not met. Again, all alpha levels in the correlation analysis were Bonferroni-corrected for multiple comparisons.

Effect sizes were measured with eta squared (η^2^) for one-way ANOVA considering large effect ≥ 0.14, medium effects ≥ 0.06, and small effects ≥ 0.01; with Cohen’s d for *t*-tests, considering large effect = 0.8; medium effect = 0.5; and small effect = 0.2; with epsilon squared (ε^2^) for Kruskal–Wallis tests considering < 0.04 = weak, 0.04–0.36 = moderate, and ≥0.36 = strong effects. The strength of associations was measured with Pearson’s (r) or Spearman’s rank (ρ) correlation coefficients, considering 0.01–0.19 = negligible, 0.20–0.29 = weak, 0.30–0.49 = moderate, 0.50–0.69 = strong, and 0.70 ≥ very strong relationships between the variables [66]. This interpretation was also applied to rank biserial correlation coefficients, which were used as effect size measures for Mann–Whitney’s U tests and to Cramer’s V values which were used for Fischer exact tests.

## 3. Results

### 3.1. Behavioural Measures

To determine behavioural differences in speech perception between the groups of children, we assessed their speech-in-babble and filtered speech perception skills. Table 1 presents the descriptive statistics for speech perception tests in the children’s groups (for data distributions, see Appendix A).

Data were normally distributed in both groups for the speech-in-babble test but not normally distributed for the filtered speech test (see Appendix B for data assumptions checks). For the speech-in-babble test, independent samples *t*-tests indicated that the difference between groups was non-significant, t(20) = 1.13, *p* = 0.272, d = 0.5 (medium effect size), despite the typical language development group showing a higher percentage of correct responses (mean = 62.5, SD = 13.43) than the developmental language disorder group (mean = 55.54, SD = 14.18). For the filtered speech test, Mann–Whitney’s U indicated that the median percent correct score was significantly higher in the typical language development (median = 16.13) than in the developmental language disorder group (median = 8.86), U = 19, z = −2.56, *p* = 0.01, with a large effect size (rank biserial correlation r = 0.66), using an exact sampling distribution for U [67].

### 3.2. Resting-State EEG Measures

#### 3.2.1. Spectral Power Analysis

- Global power.

**Figure 1:**
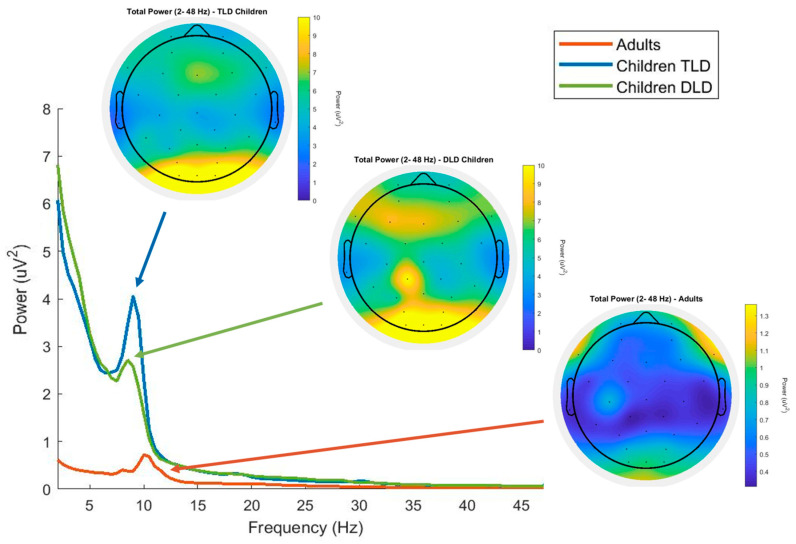
Global power spectrum (2–48 Hz) averaged across all electrodes for each group, with their respective scalp distribution map. TLD: typical language development (*n* = 11); DLD: developmental language disorder (*n* = 16); adults (*n* = 18).

As a first data check, we computed the power spectra averaged across participants for each group. In all groups, the spectrum showed the typical 1/f gradual decrease in power and the expected alpha peaks at approximately 10 Hz, as expected in a typical resting state EEG. Next, we averaged spectral power across all electrodes for each group (Figure 1). The magnitude of the signal was smaller in the adult than the children’s groups, ranging from 0.3 to 1.3 µV^2^, whereas in children the activation was in the range of 0–10 µV^2^, with similar patterns for both groups of children except in the alpha band. All three groups showed a peak in the alpha band followed by an energy decrease. In the children’s groups, the alpha peak appears slightly below 10 Hz, whereas in adults it is observed at 10 Hz. The alpha peak is larger for the typical language development than for the group with developmental language disorder. Figure 1 presents the total power scalp distribution for all groups (average from all electrodes), indicating a posterior positivity in children, and frontal-central negativities in adults, also with much larger power in the groups of children. The isolated centroparietal activation in the developmental language disorder group is likely to represent a remaining artifact.

- Average band power.

We first examined between–group differences in average power at each frequency band, calculated across 13 frontocentral electrodes (Fp1-2, AF3-4, F7-8, F3-4, FC1-2, FC5-6, and Fz). To avoid the line noise (50 Hz), a noisy electrode in the typical language development group and the filter cut-off, we restricted the range of frequencies for analysis from 2 to 45 Hz. Table 2 displays the descriptive statistics at all frequency bands for each group, evidencing two relevant features: (i) adults show smaller power than children at all frequencies, and (ii) the ratio between theta/alpha and alpha/beta is smaller in adults than in children (for data distributions, see Appendix A).

**Figure 2:**
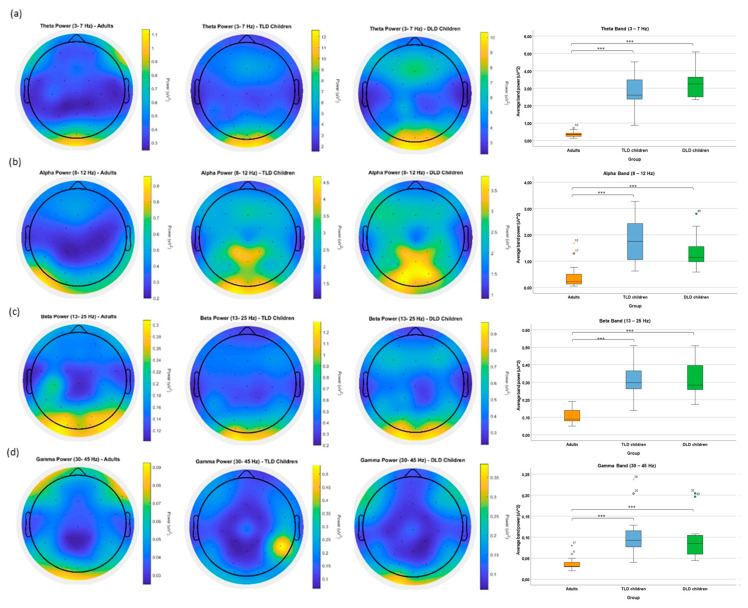
Average power for each frequency band (frontocentral electrodes). Left: Scalp maps show power distribution for the adult (left column, *n* = 18), typical language development (centre-left column, *n* = 11), and developmental language disorder (centre-right column, *n* = 16) groups. Row (**a**) theta band power (3–7 Hz); row (**b**): alpha band power (8–12 Hz); row (**c**): beta band power (13–25 Hz); and row (**d**) gamma band power (30–45 Hz). The isolated right temporoparietal activation in gamma (panel **d**) for the TLD group is likely remaining electrode noise. The colour bar scale for adults is smaller to facilitate visualisation. Right column: Box plots indicate the average band power for each group at each frequency. In all bands, power is significantly smaller for adults than children at the *p* < 0.001 level (***).

Figure 2 presents power scalp distributions for each frequency band averaged across the 13 frontocentral electrodes. Between-group planned comparisons were conducted at the four frequency bands (theta, alpha, beta, and gamma), using Bonferroni correction for multiple comparisons (corrected alpha 0.05/4 = 0.013). In general, adults showed significantly lower power than children for all frequencies, but there were no differences between the groups of children.

Panel 2a shows results for theta band power, indicating a posterior scalp distribution and stronger activation in children than in adults. One-way ANOVAs indicated a significant effect of Group (F(2,44) = 80.434, *p* < 0.001), with a large effect size (η^2^ = 0.793) and adequate power (100%). Multiple comparisons with Tamhane’s correction for unequal variances (Appendix A) indicated significantly lower theta power in adults (mean = 0.37, SD = 0.17) than in the typical language development (mean = 2.85, SD = 1.02), and in adults than in the developmental language disorder group (mean = 3.36, SD = 0.88) at the *p* < 0.001 level (95% CI [−3.359 −1.605] and [−3.582 −2.396], respectively). There was no difference in theta power between the typical language development and developmental language disorder groups (*p* = 0.476, 95% CI [−1.489 0.476]).

For the alpha band (Figure 2b), both groups of children present similar scalp patterns, consisting of broadly distributed central-posterior activations, whereas adults showed more localised, left-lateralised posterior activation. Independent-samples Kruskal–Wallis tests indicated significant between-groups differences (H (2,45) = 23.59, *p* < 0.001) with a moderate/strong effect size (ε^2^ = 0.54). Pairwise comparisons indicated significantly smaller alpha power in adults (mean rank = 11.50) than in the typical language development (mean rank = 33.00) and developmental language disorder (mean rank = 29.0) group at *p* < 0.001, but no differences between the children’s groups (*p* = 0.444).

Beta band average power and scalp distribution are presented in Figure 2c, showing comparatively greater posterior activation in adults than children, although in a smaller power scale. Again, one-way ANOVAs indicated between-group differences (F(2,44) = 32.65, *p* < 0.001) with a large effect size (η^2^ = 0.61) and adequate statistical power (100%). Multiple comparisons with Tamhane’s correction for unequal variances indicated significantly smaller beta power in adults (mean = 0.11, SD = 0.044) than in the typical language development (mean = 0.32, SD = 0.11) and the developmental language disorder group (mean = 0.32, SD = 10) at the *p* < 0.001 level (95% CI [−0.304 −0.113] and [−0.285 −0.141], respectively), with no differences between the children’s groups (*p* = 0.999, 95% CI [−0.113 0.103]).

Finally, Figure 2d displays gamma-band power, showing frontal and posterior activation in adults and broadly distributed effects in children, with a right temporoparietal focus of activation in the typical language development group, which is likely due to remaining electrode noise. Independent samples Kruskal–Wallis tests indicated significant differences between the mean ranks for the adults (10.84), typical language development (32.41), and developmental language disorder (30.09) group (H (2,45) = 25.57, *p* < 0.001) with a strong effect size (ε^2^ = 0.61). Pairwise comparisons indicated significantly smaller gamma power in adults than in children at the *p* < 0.001 level, but again with no differences between typically developing and developmental language disorder children (*p* = 0.652).

- Band ratio analysis.

An incidental finding in this study was the apparent differences in the band power ratios between our groups, which were detected after visual inspection of our data, as can be seen in the boxplots in Figure 2. Although we did not anticipate band ratios differences on our initial hypotheses, analysing these unexpected differences could uncover additional insights and provide a more comprehensive understanding of our data. Thus, we conducted a post hoc analysis to explore the observed group-level differences in the theta/alpha and theta/beta ratios.

**Figure 3:**
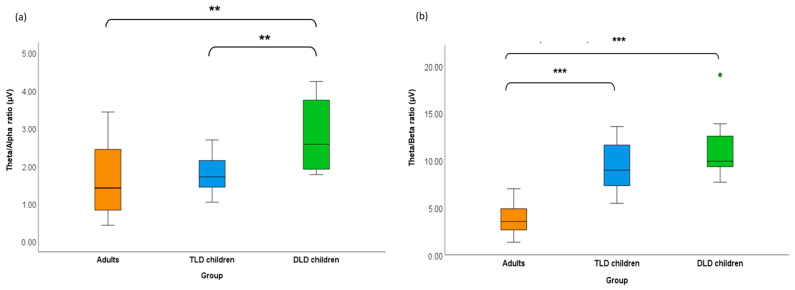
(**a**) Theta/alpha and (**b**) theta/beta ratio mean ranks for each group. (**) is significant at the *p* < 0.01 level, (***) is significant at the *p* < 0.001 level.

We compared theta/alpha and theta/beta power ratios between the groups using two independent-samples Kruskal–Wallis tests (see Appendix A for data distributions and Appendix B for normality tests) with Bonferroni-corrected alpha (0.05/2 = 0.025). Figure 3a,b display the average rank of each group for theta/alpha and theta/beta power ratios, respectively.

For the theta/alpha ratio, there was a significant difference between the mean ranks of the adult (17.5), typical language development (19.5), and developmental language disorder (31.63) groups, H (2,45) = 10.86, *p* = 0.004, with a moderate effect size (ε^2^ = 0.25). Pairwise comparisons indicated a significantly smaller theta/alpha power ratio in adults (mean = 1.63, SD = 0.97) than in the developmental language disorder group (mean = 2.82, SD = 0.96), with *p* = 0.002, and in children with typical language development (mean = 1.81, SD = 0.55) than in developmental language disorder children (*p* = 0.018). The theta/alpha ratio was smaller in adults than in the typical language development group, although this difference was non-significant (*p* = 0.697). These results suggest that adults and children with typical language skills presented a smaller theta/alpha ratio than language-impaired children.

For the theta/beta power ratio, there were significant between-groups differences in the mean ranks for adults (9.67), typical language development (28.73), and developmental language disorder (34.06) children (H (2,45) = 31.99, *p* < 001), with a strong effect size (ε^2^ = 0.73). Pairwise comparisons indicated a significantly smaller theta/alpha power ratio in adults (mean = 3.75, SD = 1.43) than in both groups of children at the *p* < 0.001 level, but no differences between the typical language development (mean = 9.27, SD = 2.66) and the developmental language disorder group (mean = 11.00, SD = 2.84), with *p* = 0.30. These results suggest that smaller theta/beta ratio could be related to the participant’s younger age, rather than their language skills.

#### 3.2.2. Hemispheric Lateralisation of Oscillations

- Average laterality indices per group.

**Figure 4:**
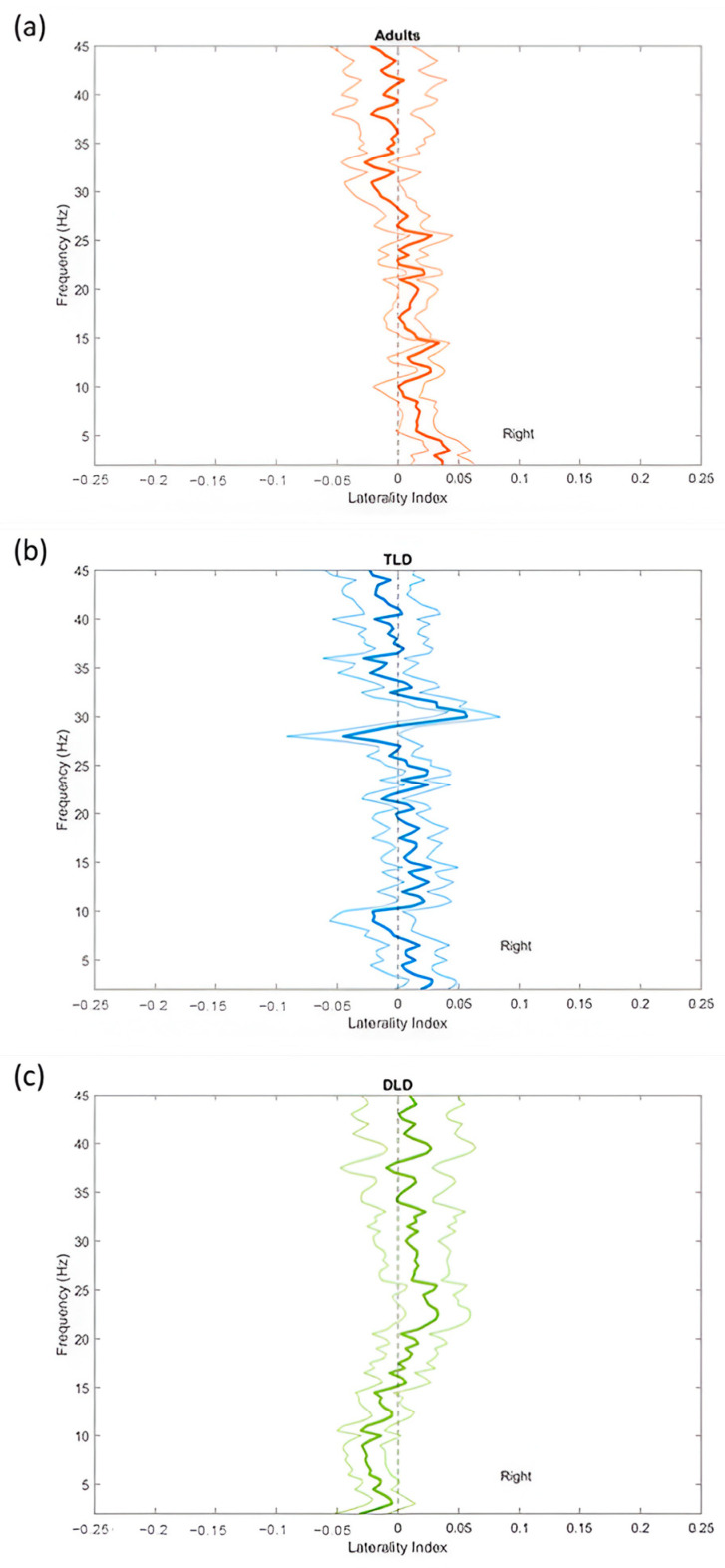
Lateralisation indices calculated at each frequency between 3 and 45 Hz for the (**a**) adults, (**b**) typically language development (TLD) and (**c**) developmental language disorder (DLD) groups. For each subplot, the area between the thin lines indicates standard error of the mean (SEM).

To determine any differences in the lateralisation of oscillations, we first computed the laterality indices at each frequency for all participants. Figure 4 illustrates the lateralisation indices at each frequency in each group. In the adult group (plot 4a), oscillations below 25 Hz are right-lateralised and slightly left-lateralised over 25 Hz. In the typical language development group (plot 4b), oscillations below 32 Hz are mostly right-lateralised, and slightly left-lateralised above that point. In the developmental language disorder group (plot 4c), oscillations below 15 Hz are left-lateralised and slightly right-lateralised between 20 and 32 Hz. The lateralisation patterns are somewhat similar between adults and children with typical language development, but the group with developmental language disorder seems different from the other two groups.

- Asymmetry of oscillations.

Laterality indices from each participant were averaged into a low-frequency bin (3–7 Hz) corresponding to the theta range and a high-frequency bin (20–45 Hz) corresponding to high beta and low gamma oscillations. Table 3 presents the descriptive statistics for laterality indices for each group (see Appendix A for data distributions). Mean values for low-frequency and high-frequency oscillations appear close to zero in all groups, suggesting no lateralisation.

Table 4 displays the results of the within-group analysis, confirming the lack of significant asymmetries in all groups. One-sample *t*-tests within each group showed that neither the low-frequency nor the high-frequency laterality indices differed significantly from zero. Likewise, paired-sample *t*-tests indicated that the differences in lateralisation indices between low and high-frequency oscillations were non-significant for all groups. These findings indicate there is no oscillatory asymmetry in any group, although with a small effect size for all the tests (d values below 0.5 for a given frequency range). For each analysis, alpha was adjusted to 0.17 to correct for multiple comparisons (0.05/3).

Lastly, we compared the lateralisation indices for low frequency and high frequency oscillations between the typical language development, developmental language disorder, and adult groups. Separate one-way ANOVAs with a Bonferroni-corrected alpha (0.17) indicated no between-group differences for low frequency [F(2,44) = 2.01, *p* = 0.147, η^2^ = 0.087] or high frequency [F(2,44) = 0.122, *p* = 0.885, η^2^ = 0.006], in both cases with small effect sizes and low statistical power (39.1% and 6.7%, respectively).

#### 3.2.3. EEG Versus Behavioural Measures

**Figure 5:**
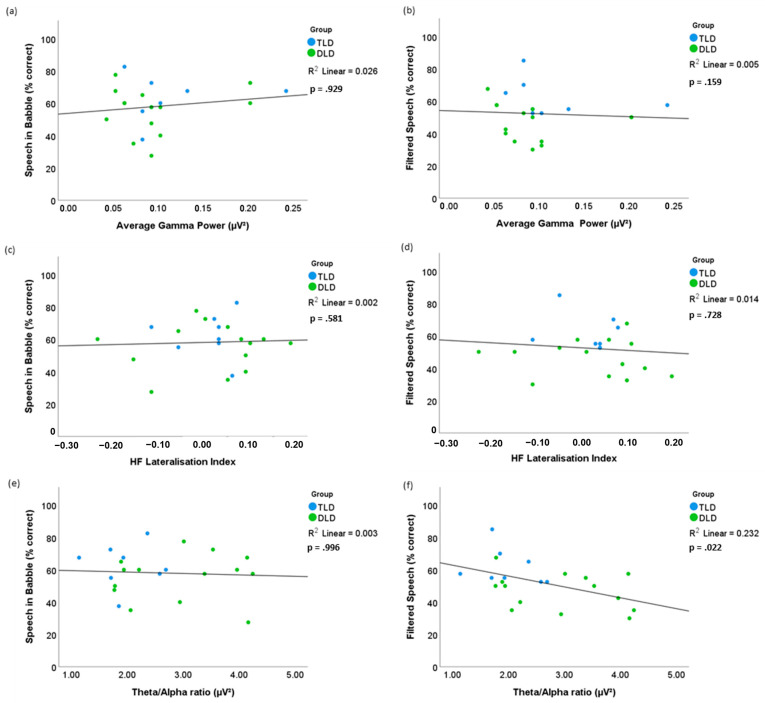
Correlation between EEG measures and speech perception performance in children: (**a**) gamma band power versus speech-in-babble, (**b**) gamma band power versus filtered speech, (**c**) high-frequency lateralisation indices versus speech in babble, (**d**) high-frequency lateralisation indices versus filtered speech, (**e**) theta/alpha ratio versus speech-in-babble, and (**f**) theta/alpha ratio versus filtered speech test results. All correlations are non-significant at the 0.008 level. TLD = typical language development. DLD = developmental language disorder.

To test the secondary hypothesis, we examined if there was an association between children’s performance in speech perception tests and the resting-state measures of interest (gamma-band power, high-frequency lateralisation indices, and theta/alpha ratio). As we had previously compared behavioural results between groups, this time we pooled all of the children together (*n* = 22), addressing the possibility that equivalent cognitive mechanisms could support speech perception performance regardless of the children’s language status. To account for non-normal distribution in most of the analysed variables (see Appendix B), in all cases, we used Spearman’s correlation tests with Bonferroni-corrected alpha = 0.05/6 = 0.008 and confidence interval (CI) estimation using the Fieller, Hartley, and Pearson methods and Fisher’s r-to-z transformation. All correlations are presented as scatterplots in Figure 5.

First, we assessed correlations between gamma band power versus the speech perception test scores. For the speech-in-babble test, there was no significant correlation (r(20) = 0.020 *p* = 0.929, 95% CI [−0.416 0.449]) between the percentage of correct answers and average gamma-band power (Figure 5a). Similarly, there was no significant correlation between the percentage of correct answers for the filtered speech test and average gamma power (r(20) = −0.311, *p* = 0.159, 95% CI [−0.655 0.140]), as shown in Figure 5b.

Then, we assessed the relationship between the high-frequency lateralisation of oscillations and speech perception performance. There was no significant correlation (r(20) = −0.125, *p* = 0.581, 95% CI [−0.529 0.325]) between the speech-in-babble test and the laterality indices for high-frequency oscillations (Figure 5c). For the filtered speech test, there was no significant correlation (r(20) = −0.079, *p* = 0.728, 95% CI [−0.494 0.366]) between the percentage of correct answers and the laterality indices for high-frequency oscillations (Figure 5d).

Finally, to examine our incidental finding about differences between the groups of children in the theta/alpha ratio, we examined the association of this measure and the speech-in-babble and filtered speech test results. For the speech-in-babble test (Figure 5e), results indicated no correlation with the theta/alpha power ratio values, (r(20) = −0.001, *p* = 0.996, 95% CI [−0.433 0.432]). Although there was a moderate negative correlation between filtered speech test scores and the theta/alpha ratio, (r(20) = −0.486, *p* = 0.022, 95% CI [−0.759 −0.086]), suggesting that detection of filtered speech is higher when the ratio between theta and alpha power is smaller, this was non-significant at the alpha-corrected level = 0.008 (Figure 5f).

## 4. Discussion

The aims of this study were twofold: (i) to examine differences in resting state EEG measures under different language skills, children with typical language development or developmental language disorder, and adults; and (ii) to investigate the association between children’s speech perception skills and high-frequency oscillations. As expected, we confirmed that adults showed significantly lower oscillatory power than children did in all the frequency ranges. However, none of the spectral power measures differed between children with developmental language disorder and their typically developing peers, providing no evidence to support our primary hypothesis of a relationship between high-frequency oscillations and language skills. Moreover, none of our groups showed a significant oscillatory asymmetry, contrary to what we expected according to previous studies and the idea of left-lateralised high-frequency oscillations acting as priming mechanism related to language processing. However, it is important to consider that the statistical power of our analysis was limited by the small sample sizes, increasing the risk of Type II errors. Thus, these null results must be interpreted carefully, as it is possible that our study was not able to detect differences in EEG measures between the groups of children (as previously reported in the literature), due to low statistical power and small effects sizes.

Regarding the differences between children and adults in spectral power, we observed similar patterns in theta, alpha, beta, and gamma bands as those described by Anderson and Perone [10], Tierney et al. [17], and Yordanova and Kolev [31]. However, we could not confirm the increase in gamma power until the age of 4 years or the decrease after this age reported by Takano and Ogawa [33]. In addition, visual inspection of our data’s global spectrum indicates differences between adults and children in the peak values at least for some of the frequency bands. We observed that the alpha peak in children occurs at slightly lower frequencies (~8 Hz) than in adults (10 Hz), which aligns with previous studies such as Kwok et al. [35]. Thus, the resting-state power patterns observed in our groups are consistent with the developmental trajectories described in the previous literature, showing an evident flattening of spectral power between early childhood and adulthood. In resting-state EEG literature, greater alpha and beta power has been associated with increased arousal and top-down attentional control [68] which, in our study, could indicate greater alertness and wakefulness in adults than children. Although the functional interpretation of resting-state theta power is less clear, recent studies suggest that it reflects cognitive control processes, and may increase during excessive monitoring, shifting, or updating operations [69]. Another possible speculation is that age-related reduction in theta power could reflect brain maturation and more efficient network synchronisation. These developmental changes could lead to adult-like local and long-range theta connectivity through more consistent phases, rather than amplitude signalling. Future studies could use functional connectivity analysis to elucidate these points.

Regarding lateralisation of resting-state oscillations, we found no significant hemispheric asymmetry for the oscillation frequency ranges related to phonemic (high frequency) or syllabic processing (low frequency) in any of our groups, meaning that we found no evidence in our groups to support the Asymmetric Sampling in Time hypothesis. For the low-frequency range, our findings are congruent with those of Thompson et al. [51], who reported centrally distributed low-frequency oscillations with similar mean group indices (−0.002). However, we did not observe the high-frequency leftward bias reported in their study, which we would have expected, at least in the adult group, according to the Asymmetric Sampling in Time hypothesis. A possible alternative explanation is that the effect sizes for lateralisation in our study (less than d = 0.5) are smaller than in Thompson’s (d = 1.14), which could have prevented us from detecting any potential asymmetry in our groups, increasing the possibility of Type II error. Alternatively, the lack of lateralised oscillatory patterns may suggest that speech perception does not always rely on strict left-hemisphere dominance, as previously proposed by Wilson and Bishop [56], or at least, not in resting-state conditions. The absence of hemispheric asymmetry and bilateral activation suggests that, at rest, oscillations involved in speech perception may not be primed for phonemic (e.g., high-frequency oscillations dominating in the left hemisphere) or prosodic processing (e.g., low-frequency oscillations dominating in the right hemisphere), as proposed by the Asymmetric Sampling in Time Hypothesis. Although our findings do not align the conventional understanding of hemispheric lateralization in speech perception, it is noteworthy that resting-state activity may differ from brain dynamics during speech perception, explaining the discrepancy between endogenous and task-related oscillatory activity. However, there are remaining questions about the developmental trajectories of oscillatory asymmetries and potential compensatory processes in atypical populations.

Importantly, we must consider EEG methodological aspects when interpreting the lateralisation results. For example, to determine asymmetry we used average measures at each frequency range; in the future, performing point-by-point *t*-tests or permutation tests instead of conventional *t*-tests could improve the sensitivity to detect clusters of significantly lateralised oscillations. Secondly, to avoid electric noise and artifacts in our data, we defined the range of high-frequency oscillations between 25 and 45 Hz, whereas Benasich et al. [45], Gou et al. [34], and Thompson et al. [51] used 30–51 Hz. This shift in high-frequency boundaries could have affected our results by introducing more high-beta and less high-gamma band activity in our high-frequency range. Thirdly, lateralisation indices are a power-based measure; thus, if spectral power shows age-related reduction, it may not be appropriate to compare adult and children lateralisation indices directly without some scaling or normalisation procedure.

An unexpected finding of this study was the significant differences in the band ratio measures, especially the differences in theta/alpha band power ratios between the children’s groups. Interestingly, this was the only measure that was consistent with our group’s typical/atypical developmental language status. Previous evidence suggests that band ratios are reliable indicators of cognitive performance [70,71], and may even serve as clinical biomarkers of cognitive dysfunction across various neuropsychiatric disorders, including dementia, attention deficit hyperactivity disorder (ADHD), Alzheimer’s and Parkinson’s diseases (e.g., [72,73,74]), although these measures have not been studied in developmental language disorder. Specifically, we found that theta/alpha band ratios were smaller in the groups with better language skills; this is, in adults and typically developing children than in the developmental language disorder group. Theta/alpha ratio was the only EEG measure that differed between typical/atypical language development. Although non-significant after correcting alpha levels for multiple comparisons to 0.008, the theta/alpha ratio also tended to inversely correlate with children’s filtered speech test scores, which would align with findings by Kwok et al. [35] showing an inverse correlation between alpha power and language skills in children at similar ages. For the theta/beta ratio, values were significantly smaller in adults than in children, but with no difference between the groups of children, suggesting that theta/beta ratio could be sensitive to age differences but not to the group’s language status. However, although post hoc analyses are valuable, as they allow us to investigate patterns that were not considered in the original study, it is essential to interpret band ratio findings cautiously; to consider them a confirmation of the relationship between resting-state activity and language skills or age, we should control for other untested cognitive variables related with theta/beta and theta/alpha band ratios; for example, the effects of non-verbal or attentional abilities. Nevertheless, our band ratio findings could be used in the generation of future hypotheses.

That being said, the band ratio results in this study indicate that developmental changes in the resting-state EEG involve a global reduction in spectral power, but this reduction may not be uniform for each frequency band. For example, the band-ratio differences in our groups could reflect an age-related decrease in theta band power, but less decrease in alpha or beta power. However, the functional significance of EEG band power ratios has not been established yet. Some studies indicate that band ratio measures reflect periodic and aperiodic spectral activity, which could conflate power measures, leading to incorrect interpretations [44]. On the contrary, other studies propose the beta/theta ratio as a marker of cognitive processing capacity, which could be modulated by age. For example, Tramell et al. [75] showed that theta/alpha ratio was related to cognitive abilities and modulated by age in young (below 30 years) and older (over 70 years) neurotypical adults. Higher frontal alpha and lower theta power (resulting in a low theta/alpha ratio) seems to predict inhibitory control in middle childhood [9], while the opposite pattern appears in children who stutter [76] and those with learning disabilities [77] compared with typically developing peers. In addition, resting-state theta/beta ratio is also associated with executive functions in typically developing children [9,32] and infants [78], which is important to consider given that executive functions and verbal abilities are often correlated [78]. For example, research by Zivan et al. [79] in typically developing children found an inverse correlation between theta/beta ratio and reading accuracy levels, interpreting these results as reflecting different levels of attentional demands rather than language processing. This indicates that the question about the origin of band ratio differences remains open and needs to be addressed in future studies to determine their functional significance and define their developmental trajectories.

For our secondary hypothesis, we partially confirmed previous findings (e.g., [80]), of better speech perception performance in children with typical language development than in those with developmental language disorder. However, we only observed such differences for the filtered speech, and not for the speech-in-babble test, despite previous reports to the contrary (e.g., [81]). Our results were not explained by differences in the children’s hearing levels, age, gender, or SES, because these were equivalent between groups. Moreover, a recent, large-scale EEG study [82] found that the association between frontal gamma power and late talker status is not moderated by demographic factors such as gender, SES (indexed by maternal education), or ethnicity, suggesting that these demographic variables may be less influential than previously thought for studies relating language outcomes and EEG measures. However, these differences may have to do with other differences between the typical language development and developmental language disorder groups; for example, in phonological processing (see [41,58]) or more general cognitive skills, such as arousal or attention levels. Importantly, the speech tests we used in this study evaluate different aspects of speech processing, which may have distinct neural substrates. Speech-in-babble tests assess the ability to separate spoken words from the background, which requires selective attentional processing. At the neural level, this ability results from an enhancement of task-relevant neural activity (target speech stimuli) and inhibition of responses to non-relevant speech stimuli [83], which could be preserved in both groups of children, as developmental language disorder is not essentially an attentional deficit. On the other hand, filtered speech requires the brain to fill in a degraded speech signal, which is more related to priming or top-down modulations by previous language knowledge, including phonological and lexical skills. Phonological processing deficits are a common symptom of developmental language disorder [41] and may result in a decreased capacity in these children to discriminate words in an acoustically degraded speech signal in comparison with typically developing controls, as was reported by Goswami et al. [84]. Thus, is it possible that different speech processing skills could develop differently in children with typical language development and developmental language disorder explaining the difference in performance only for the filtered speech test.

In addition, we did not find the expected association between speech perception performance and resting state EEG measures. This does not align with previous findings by Benasich et al. [45], Gou et al. [34], and Thompson et al. [51], indicating that the majority of our resting state measures of cortical activity do not reflect our participant’s language status (adult-like, typical language development, or developmental language disorder), or the children’s speech perception skills. As resting-state power measures were similar in both groups of children, the differences we observed in band power between children and adults are more likely a reflect brain maturation (e.g., an effect of age), and not an effect of language typical/atypical status. Importantly, it is worth considering that the resting-state measures used in our study may not be precise or sensitive enough to inform meaningful conclusions about language processing in typical and atypical language development.

Chiefly, our results are inconsistent with previous findings about a positive association between resting-state frontal gamma power and language development [34,55] as we found no association between frontal gamma power and typical/atypical language status. Our results could be explained by the fact that participants in previous studies were younger than in ours, and their age range was considerably broader. Benasich et al. [45] and Gou et al. [34] studied infants aged 6 to 36 months, so it is possible that the differences in gamma power they reported will have already disappeared or be harder to detect by the age of our groups (4.6–5.7 years). Another possible explanation is that, despite children’s typical/atypical language status, there is still heterogeneity in language skills between children at the same age, even within clinical and control groups, making any group-level differences in gamma power harder to detect. A feasible way to examine in more depth the association between language development and resting state measures would be to re-test our children’s groups in the future and see whether the gamma power recorded in the current study predicts their later language skills.

Overall, this study’s main contribution is characterising resting-state EEG activity in preschoolers with typical language development and those with a clinical diagnosis of developmental language disorder. So far, we are unaware of any research examining power and lateralisation measures in these groups between the ages of 4 and 6 years and at the same time investigating their relationship with speech perception measures. Pursuing this line of research is relevant for developmental cognitive research, as by understanding and monitoring children’s resting-state EEG activity, researchers and clinicians may be able to identify those who are at risk for neurodevelopmental disorders (e.g., developmental language disorder), and provide early intervention to support them. However, it is important to consider that our study has some limitations.

The main limitation is the small sample size of our groups of children, which was further reduced due to some children not attending the third session for behavioural testing. The final sample sizes in the developmental language disorder and control groups limit the statistical power if the analyses and increase the possibility of Type II errors. This reduces the strength of our findings, particularly those about the relationship between EEG measures and performance in the speech perception tests. Initially, our plan was to include 25 children per group to match their size more closely with the adult group, which was part of a previous validation study. However, due to participant drop-out and rejection (e.g., due to not meeting the study criteria or failing screenings), our sample size in December 2019 was reduced to the current one. At the time, we planned to collect data from new children during 2020, but it was impossible due to the COVID-19 pandemic. For the next two years after initial data collection, we were not able to travel to Chile (because of international travel restrictions) or bring children to the lab there (because of ethics restrictions), meaning the second data collection phase had to be cancelled. In the future, more studies must confirm these results in a larger, more representative sample of children and adults.

Another aspect to consider is that the participants in the adult group were not Spanish monolinguals, but had different levels of proficiency and age of acquisition of English as their second language, which may confound the effects of age the effects of bilingualism on resting-state power measures, which have been reported in the literature (for example, see [84]). Although monolingual adults from Chile would have been the optimal adult group, this was not logistically possible for this study, as it required additional data collection overseas. However, in the group of Chilean adults we included from our previous study, Spanish was the dominant language, with a higher frequency of use than English. Moreover, none of the adults had lived permanently in an English-speaking country or were early bilinguals. We believe these characteristics could reduce the potential effects of bilingualism in their resting-state activity, making them more alike to Chilean Spanish monolingual speakers (or monolingual with an L2, as in [84]). In addition, an important consideration that may help to disentangle the effects of brain maturation from those of bilingualism on resting-state power is that bilingual individuals exhibit higher alpha and beta power than monolinguals, but only in right posterior electrodes, as reported by Bice et al. [85]. Therefore, our results may actually show the effects of age, and not of bilingualism, as our analysis only included frontocentral electrodes, in which no such effects have been detected [85].

In terms of the EEG methods, another limitation is that the band power and laterality measures used in this study are both based on the amplitude of the spectral energy, which is affected by individual variation in EEG, neural noise, small sample size, and small effect sizes, especially when using group average values. Thus, future research could investigate other resting state EEG measures that are less reliant on spectral power. For example, functional connectivity analysis examines the degree of synchronisation of neural populations between different brain regions, providing information about large-scale brain dynamics at rest. Thus, combining spectral power measures and functional connectivity analyses could offer complementary insights into resting-state brain activity and its relationship with language and cognition. Notably, there is growing research interest in the role of aperiodic (arrhythmic) electrophysiological activity, which is abundant in children’s EEG measures and reflects excitatory and inhibitory balance in cortical networks (see Ostlund et al. for a toolbox and tutorial [21]). Including aperiodic measures in the parameterisation of the neural power spectra (e.g., aperiodic offsets and exponents) and functional connectivity measures could contribute to more accurate descriptions of cognitive and language development in young children [44].

Importantly, the behavioural tests in this study may not have reflected children’s speech perception performance in their daily lives. As with many clinical measures, the speech perception tests consisted of isolated words, so it could be argued that they primarily reflect auditory rather than speech perception skills, as at the single word level, speech processing cannot be modulated by linguistic processing or language skills (e.g., as predictive processing or the use of linguistic context). Future studies could use more ecological behavioural measures, such as the perception of sentences or continuous speech.

Finally, it is possible that other cognitive factors than language skills may have influenced the resting-state EEG activity, such as the participant’s arousal, attention, or wakefulness level. Despite that we monitored these factors during EEG acquisition according to standard procedures in EEG research, which are especially important when testing young children [65], we cannot rule out that fatigue, boredom or inattention may have influenced the participant’s resting-state EEG patterns, especially in our groups of preschoolers. The lack of more robust control for this factor is an important limitation that could be addressed in future studies, as it could potentially explain the lack of differences in resting-state measures between the groups with typical and atypical language development.

## 5. Conclusions

Our findings confirm that EEG resting-state activity is stronger in children than in adults, but do not support the idea of differences in spectral power between the typical language development and developmental language disorder groups. Importantly, the lack of significant oscillatory asymmetry in all our groups and the lack of correlation between speech perception measures and high-frequency/gamma oscillatory activity does not align with previous findings. Furthermore, our study did not find any evidence to support previous theories, such as the Asymmetric Sampling in Time Hypothesis, or the idea of atypical brain lateralisation in developmental language disorder. We also observed that the ratio between theta and alpha band power was significantly different between the groups of preschoolers with typical/atypical language development, suggesting that this ratio may be a more relevant index for language skills compared to independent theta and alpha power. Future studies could further explore the functional significance of this measure.

## Figures and Tables

**Table 1:** Descriptive statistics for speech perception tests, in both groups of children.

	Typical Language Development *n* = 8		Developmental Language Disorder *n* = 11	
Test	*Mean*	*SD*	*Mean*	*SD*
Speech-in-babble	62.50	13.43	55.54	14.18
Filtered speech	61.56	11.33	46.79	11.16

*Note.* Missing values excluded.

**Table 2:** Descriptive Statistics for average band power (µV^2^) per frequency band.

		Typical Language Development *n* = 11		Developmental Language Disorder *n* = 16		Adults *n* = 18	
	Hz	*Mean*	*SD*	*Mean*	*SD*	*Mean*	*SD*
Theta	3–7	2.85	1.02	3.36	0.88	0.37	0.17
Alpha	8–12	1.73	0.86	1.32	0.57	0.41	0.44
Beta	13–25	0.32	0.11	0.32	0.10	0.11	0.04
Gamma	25–45	0.11	0.06	0.09	0.05	0.04	0.12

**Table 3:** Descriptive statistics for laterality indices.

		Typical Language Development *n* = 11		Developmental Language Disorder *n* = 16		Adults *n* = 18	
	Hz	*Mean*	*SD*	*Mean*	*SD*	*Mean*	*SD*
Low-frequency	3–7	0.01	0.07	−0.02	0.06	0.03	0.06
High-frequency	20–45	0.001	0.06	0.013	0.11	−0.002	0.09

**Table 4:** Within-groups analysis for laterality/asymmetry measures.

Group	Asymmetry Measure	*t*-Test Type (One Sided)	df	t	*p*	Cohen’s d
Typical language development	LF vs. zero	One sample	10	0.501	0.314	0.151
	HF vs. zero	One sample	10	0.048	0.481	0.015
	LF vs. HF	Paired sample	10	0.491	0.317	0.148
Developmental language disorder	LF vs. zero	One sample	15	−10.17	0.130	−0.292
	HF vs. zero	One sample	15	0.459	0.326	0.115
	LF vs. HF	Paired sample	15	−10.233	0.118	−0.308
Adults	LF vs. zero	One sample	17	10.84	0.04	0.43
	HF vs. zero	One sample	17	−0.09	0.46	0.09
	LF vs. HF	Paired sample	17	10.15	0.133	0.11

*Note.* LF: low-frequency oscillations; HF: high-frequency oscillations. Bonferroni-corrected alpha = 0.017.

**Figure A1:**
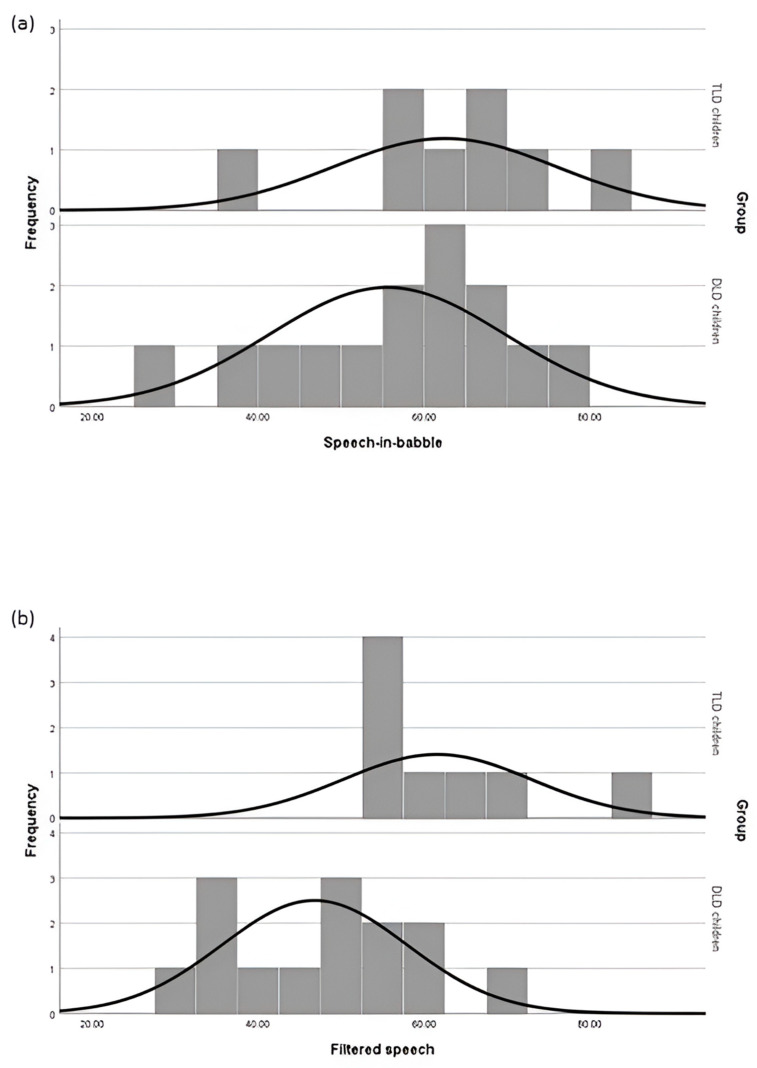
Histograms for speech perception tests scores: (**a**) speech-in-babble; (**b**) filtered speech. TLD children: typically developing group; DLD children: developmental language disorder group.

**Figure A2:**
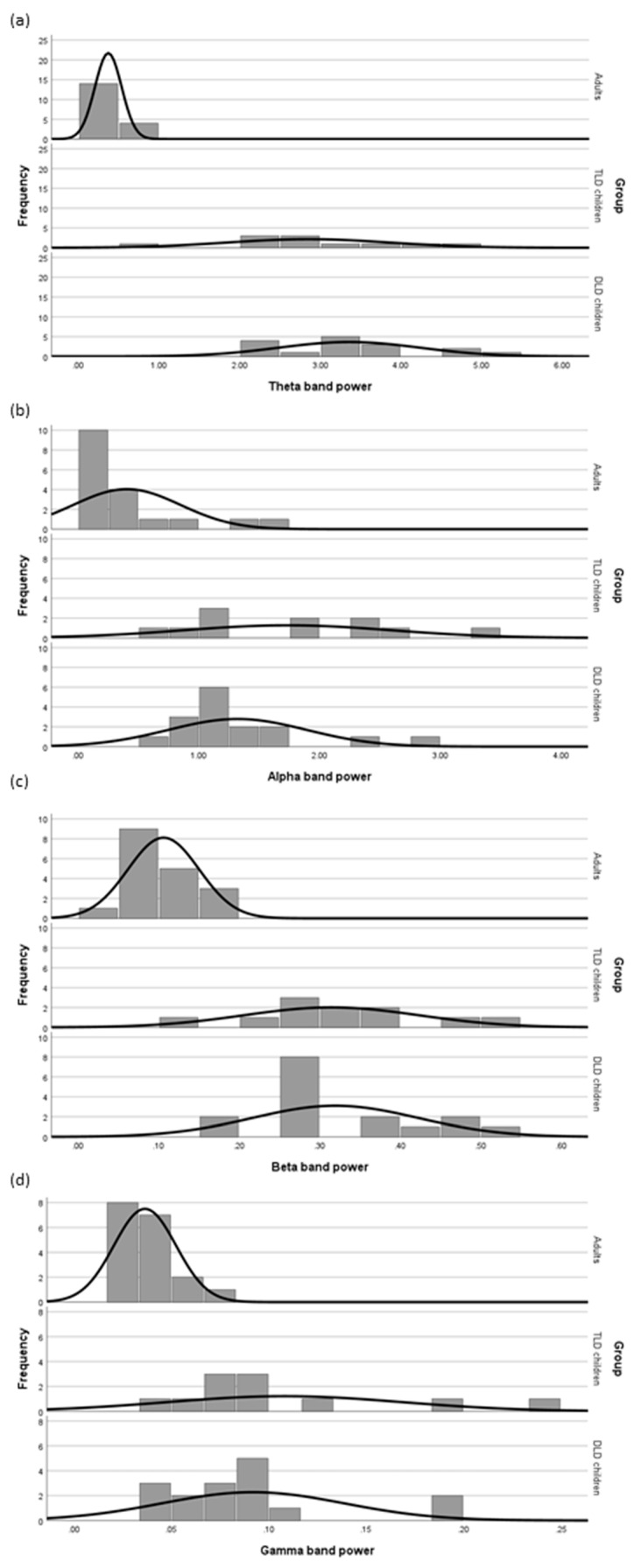
Histograms for frequency band power: (**a**) theta, (**b**) alpha, (**c**) beta, and (**d**) gamma. TLD children: typically developing group; DLD children: developmental language disorder group.

**Figure A3:**
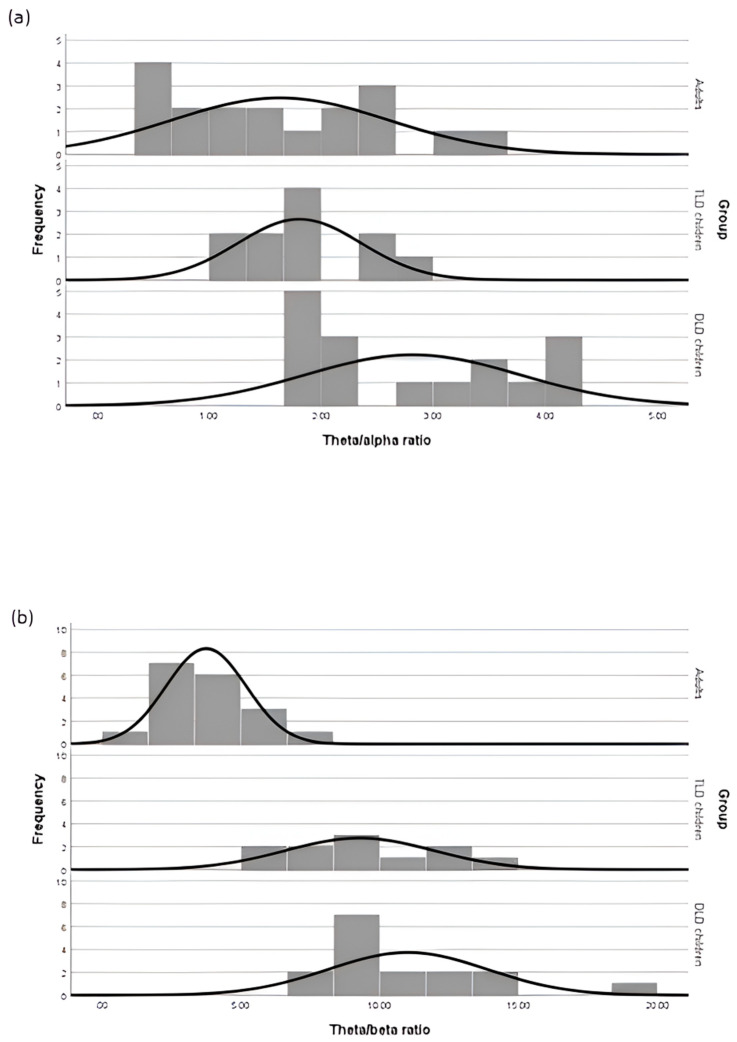
Histograms for (**a**) theta/alpha power ratio; (**b**) theta/alpha power ratio. TLD children: typically developing group; DLD children: developmental language disorder group.

**Figure A4:**
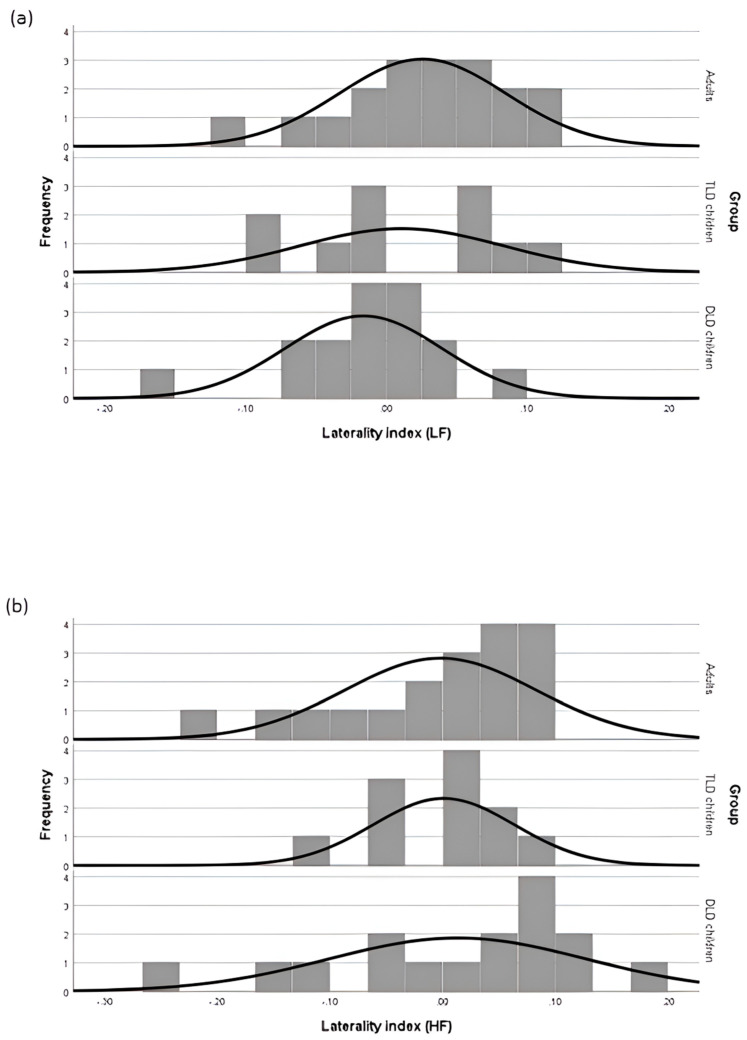
Histograms for the laterality index values at (**a**) low frequencies (LF); (**b**) high frequencies (HF). TLD children: typically developing group; DLD children: developmental language disorder group.

**Figure A5:**
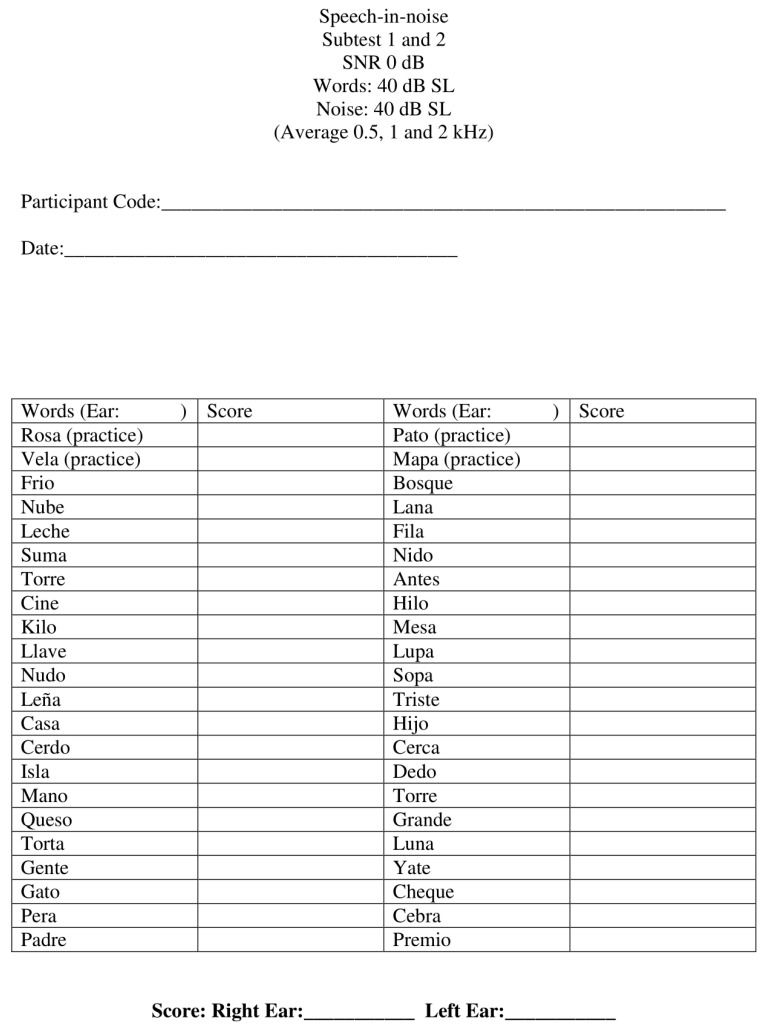
Speech-in-babble test stimuli.

**Figure A6:**
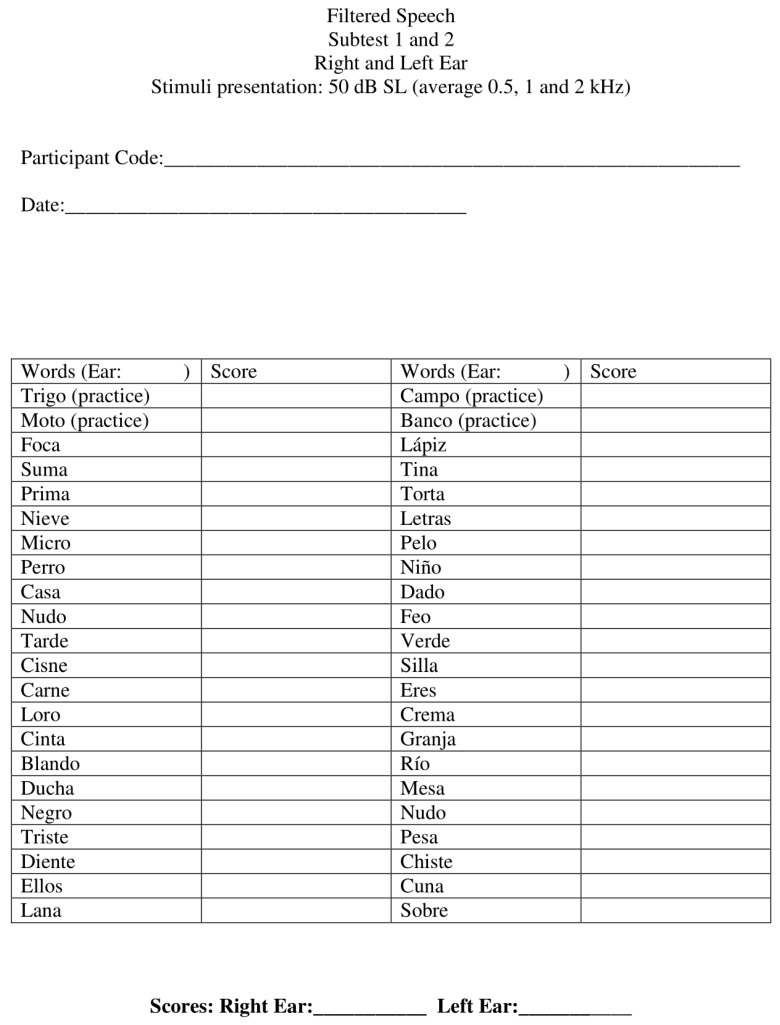
Filtered speech test stimuli.

**Figure A7:**
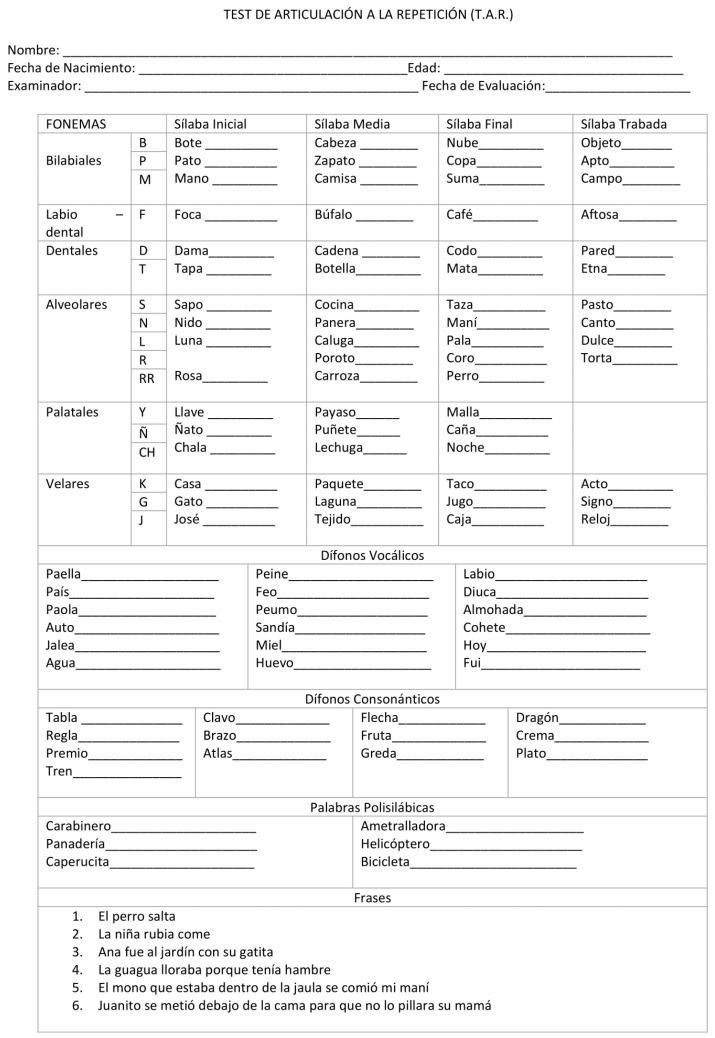
Articulation screening test (T.A.R) form.

## Data Availability

The data generated and analysed in this study are available on reasonable request from the corresponding author. The data are not publicly available as they are human data from adults and children in neurotypical and clinical groups.

## Supplementary Materials

The following supporting information can be downloaded at: https://www.mdpi.com/article/10.3390/brainsci15030219/s1, Figure S1: Distribution of the screening variables for each participant’s group; Figure S2: Example EEG waveforms for each participant’s group; Table S1: Descriptive statistics and normality test for screening variables; Table S2: Contingency tables for the variables ‘Group’ and ‘Gender’; Table S3: Analysis of association between the variables ‘Group’ and ‘Gender’ (Fisher’s exact test); Table S4: Contingency tables for the variables ‘Group (children)’ and ‘EEG Timeslot’; Table S5: Analysis of association between the variables ‘Group (children)’ and ‘Timeslot’ (Fisher’s exact test).

## Appendix A

Data distributions for speech perception tests.

Data distributions for frequency band power.

Data distributions for band power ratio values.

Data distributions for laterality indices.

## Appendix B

**Table A1:** Normality and variance homogeneity tests for the study variables.

		Test						
		Normality			Homogeneity of Variances			
Variable	Group	Shapiro–Wilk Statistic	df	*p*	Levene Statistic	df1	df2	*p*
Theta Band Power	Adults	0.936	18	0.246	9.285	2	42	**<0.001**
	TLD children	0.973	11	0.917				
	DLD children	0.892	16	0.059				
Alpha Band Power	Adults	0.737	18	**<0.001**	4.187	2	42	**0.022**
	TLD children	0.934	11	0.456				
	DLD children	0.866	16	**0.023**				
Beta Band Power	Adults	0.932	18	0.214	5.606	2	42	**0.007**
	TLD children	0.967	11	0.859				
	DLD children	0.91	16	0.118				
Gamma Band Power	Adults	0.795	18	**0.001**	5.133	2	42	**0.01**
	TLD children	0.826	11	**0.02**				
	DLD children	0.797	16	**0.002**				
Laterality Index LF	Adults	0.933	18	0.224	1.275	2	42	0.29
	TLD children	0.945	11	0.576				
	DLD children	0.901	16	0.083				
Laterality Index HF	Adults	0.912	18	0.093	1.941	2	42	0.156
	TLD children	0.875	11	0.091				
	DLD children	0.945	16	0.42				
Theta/Alpha Ratio	Adults	0.927	18	0.173	4.54	2	42	**0.016**
	TLD children	0.946	11	0.597				
	DLD children	0.853	16	**0.015**				
Theta/Beta Ratio	Adults	0.971	18	0.813	2.801	2	42	0.072
	TLD children	0.95	11	0.647				
	DLD children	0.865	16	**0.023**				
Speech-in-Babble	TLD children	0.967	8	0.872				
	DLD children	0.96	14	0.718				
Filtered Speech	TLD children	0.816	8	**0.043**				
	DLD children	0.951	14	0.572				

*Note.* TLD: typically developing group; DLD: developmental language disorder group. Bold fonts indicate test is significant at the *p* = 0.05 level (non-normal data distribution).
