# Supplementary material for: Resting-State Electroencephalogram and Speech Perception in Young Children with Developmental Language Disorder
Source: Brain Sci. 2025 Feb 20;15(3):219. doi: 10.3390/brainsci15030219 (PMC11940439; doi:10.3390/brainsci15030219)
Supplement: Supplementary file 1 [file brainsci-15-00219-s001.zip › brainsci-3382034-supplementary.pdf]

Supplementary Materials

Table S1

Descriptive statistics and normality test for screening variables

	Group	**Age at EEG (decimal)**	**PTA right** (dB HL)	**PTA left** (dB HL)	**PTA average** (dB HL)
Mean	Adults	33.68	5.94	6.56	6.25
	TLD children	5.19	21.25	20.91	21.08
	DLD children	5.16	20.63	20.55	20.59
Median	Adults	32.95	7.00	6.00	6.50
	TLD children	5.25	21.25	20.00	20.60
	DLD children	5.08	20.00	20.00	20.00
Standard deviation	Adults	4.79	3.83	3.60	3.54
	TLD children	0.26	1.58	1.69	1.53
	DLD children	0.33	1.12	0.91	0.82
Minimum	Adults	24.90	0.00	0.00	0.00
	TLD children	4.80	20.00	20.00	20.00
	DLD children	4.75	20.00	20.00	20.00
Maximum	Adults	44.11	13.00	13.00	13.00
	TLD children	5.50	25.00	25.00	23.80
	DLD children	5.58	23.75	22.50	22.50
Shapiro-Wilk W	Adults	0.96	0.91	0.96	0.98
	TLD children	0.89	0.80	0.62	0.71
	DLD children	0.85	0.64	0.64	0.76
Shapiro-Wilk p	Adults	0.623	0.086	0.622	0.964
	TLD children	0.143	**0.008**	**< .001**	**< .001**
	DLD children	**0.015**	**< .001**	**< .001**	**< .001**

Note. TLD: Typically developing group; DLD: Developmental language disorder group. Bold fonts indicate the test is significant at the p = 0.05 level (non-normal data distribution).

Table S2

Contingency tables for the variables ‘Group’ and ‘Gender’

	Gender		
Group	Male	Female	Total
Adults	7	11	18
TLD children	4	7	11
DLD children	10	6	16
Total	21	24	45

Table S3

Analysis of association between the variables ‘Group’ and ‘Gender’ (Fisher’s exact test)

	Value	df	p
χ^2	2.52	2	0.284
Fisher's exact test			0.313
N	45		

Table S4

Contingency tables for the variables 'Group (children)' and 'EEG Timeslot'

	EEG Time slot						
Group	9:30-11:30	11:30-13:30	13:30-15:30	14:00-16:00	15:30-17:30	16:00-18:00	Total
TLD children	4	4	0	2	0	1	11
DLD children	4	4	1	2	2	3	16
Total	8	8	1	4	2	4	27

Table S5

Analysis of association between the variables 'Group (children)' and 'Timeslot' (Fisher's exact test)

	Value	df	p
χ^2	3.18	5	0.672
Fisher's exact test			0.827
N	27		

Figure S1

Distribution of the screening variables for each participant's group

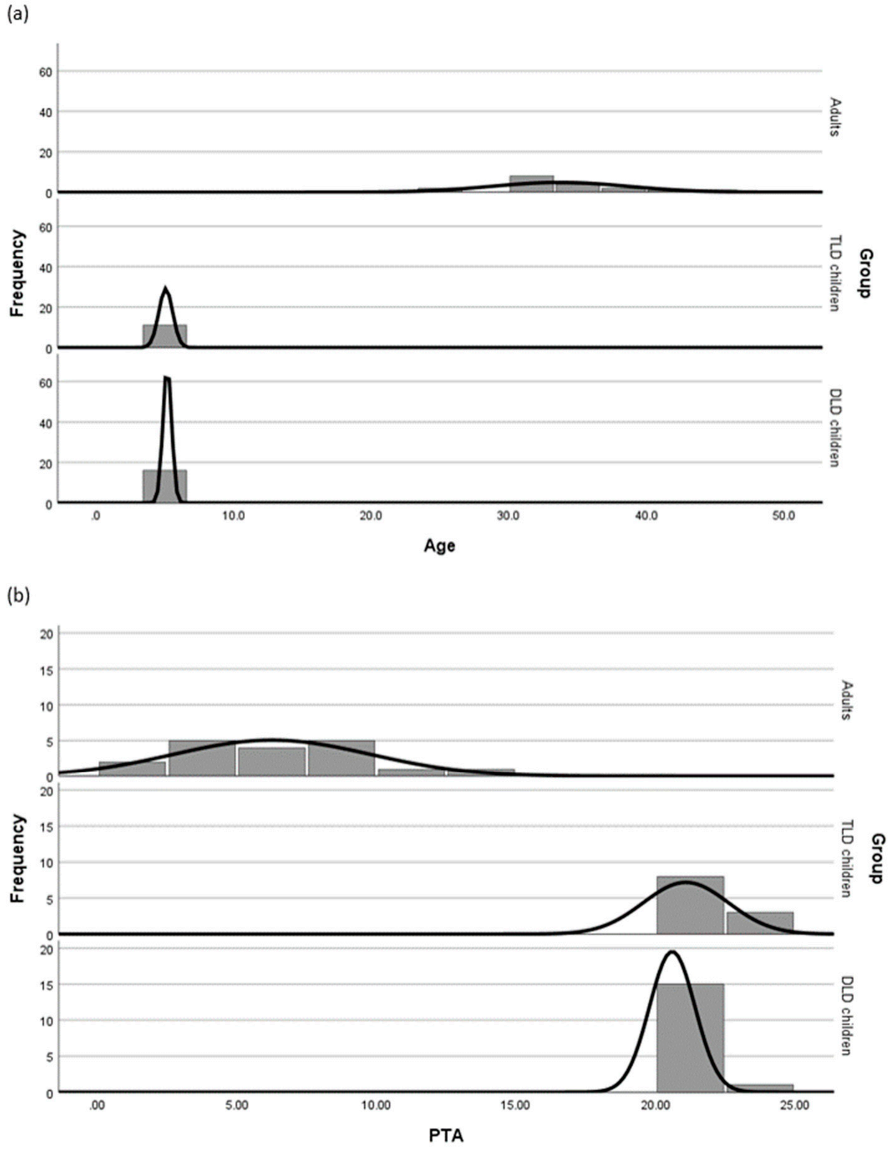

Note: Histograms for (a) Age (converted to decimal values); (b) Pure tone average (PTA). TLD children: Typically developing group; DLD children: Developmental language disorder group.

Figure S2

Example EEG waveforms for each participant's group

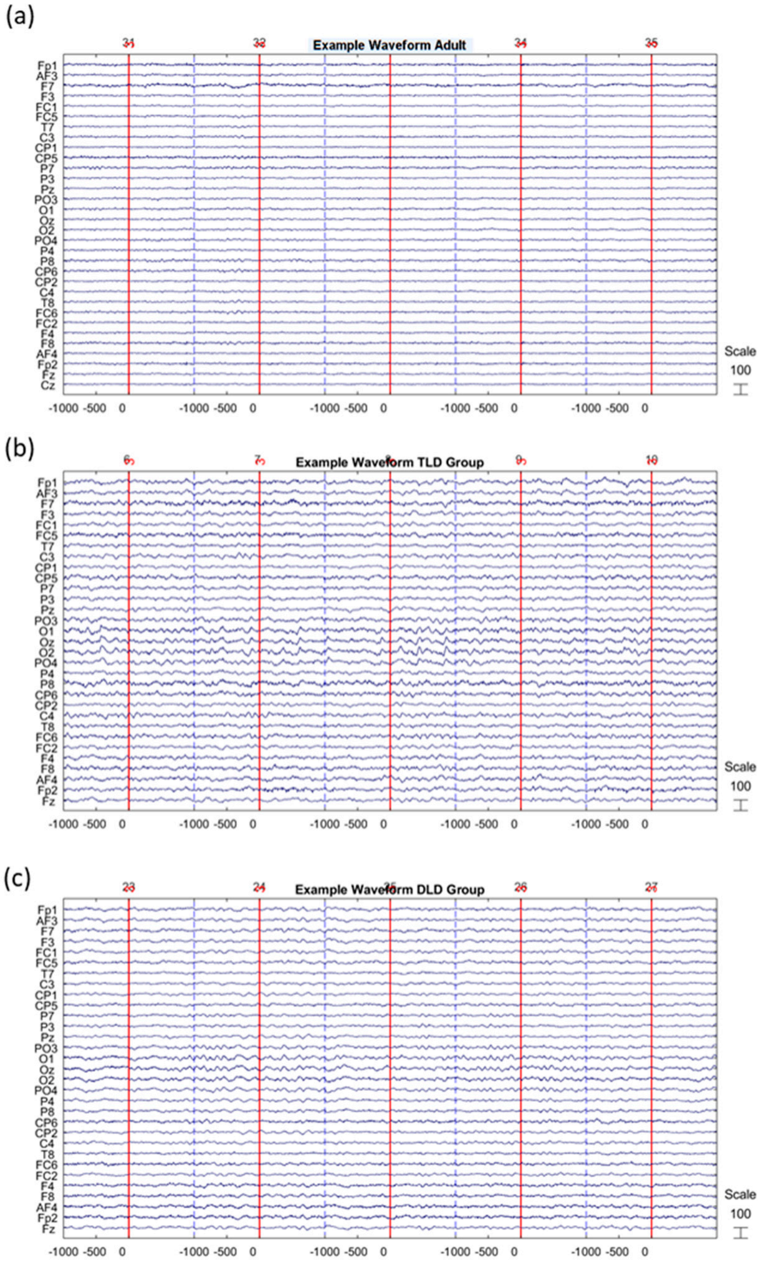

Note: Example EEG waveforms after data cleaning for a single participant in the (a) Adult, (b) Typical language development, TLD and (c) Developmental language disorder group. Red vertical lines indicate time = 0, blue vertical lines indicate epochs limits.
